# Neurobiological Mechanisms of Electroconvulsive Therapy: Molecular Perspectives of Brain Stimulation

**DOI:** 10.3390/ijms26125905

**Published:** 2025-06-19

**Authors:** Ermin Fetahovic, Vladimir Janjic, Maja Muric, Nemanja Jovicic, Branimir Radmanovic, Gvozden Rosic, Dragica Selakovic, Milos Filipovic, Nemanja Muric

**Affiliations:** 1 Department of Communication Skills, Ethics, and Psychology, Faculty of Medical Sciences, University of Kragujevac, 34000 Kragujevac, Serbia; erminfetahovic96@gmail.com; 2 Department of Psychiatry, Faculty of Medical Sciences, University of Kragujevac, 34000 Kragujevac, Serbia; biokg2005@yahoo.com (B.R.); nmuric91@gmail.com (N.M.); 3 Psychiatric Clinic, University Clinical Center Kragujevac, 34000 Kragujevac, Serbia; 4 Department of Physiology, Faculty of Medical Sciences, University of Kragujevac, 34000 Kragujevac, Serbia; majanikolickg90@gmail.com (M.M.); grosic@fmn.kg.ac.rs (G.R.); dragica984@gmail.com (D.S.); 5 Center of Excellence for the Study of Redox Balance in Cardiovascular and Metabolic Disorders, University of Kragujevac, 34000 Kragujevac, Serbia; 6 Department of Histology and Embryology, Faculty of Medical Sciences, University of Kragujevac, 34000 Kragujevac, Serbia; nemanjajovicic.kg@gmail.com; 7 Doctoral Academic Studies-Medical Sciences, Faculty of Medical Sciences, University of Kragujevac, 34000 Kragujevac, Serbia; milosfilipovic88@hotmail.com

**Keywords:** electroconvulsive therapy, neurobiological mechanisms, neurotrophic factors, neuroplasticity, oxidative stress

## Abstract

Electroconvulsive therapy (ECT) remains one of the most effective interventions for treatment-resistant psychiatric disorders, particularly major depressive disorder and bipolar disorder. Despite extensive clinical and preclinical investigations, the precise neurobiological mechanisms underlying ECT’s therapeutic effects are not fully understood. This review explores the molecular and cellular pathways involved in ECT, emphasizing its impact on neurotrophic signaling, oxidative stress, apoptosis, and neuroplasticity. Evidence suggests that ECT modulates brain-derived neurotrophic factor and other neurotrophic factors, promoting synaptic plasticity and neuronal survival. Additionally, ECT influences the hypothalamic–pituitary–adrenal axis, reduces neuroinflammation, and alters neurotransmitter systems, contributing to its antidepressant effects. Recent findings also highlight the role of mitochondrial function and oxidative stress regulation in ECT-induced neural adaptation. By synthesizing current molecular insights, this review provides a comprehensive perspective on the neurobiological mechanisms of ECT, offering potential directions for future research and therapeutic advancements in brain stimulation.

## 1. Introduction

Major depressive disorder (MDD) is a complex psychiatric condition defined by at least one depressive episode lasting a minimum of two weeks. The primary clinical features of MDD include a persistently depressive mood or anhedonia, accompanied by various neurocognitive and neurovegetative symptoms, such as impaired concentration, changes in sleep patterns, and other disturbances in physiological functioning [1]. Globally, it is estimated that approximately 280 million individuals are affected by depression [2], with a higher prevalence observed in women compared to men [3]. In 2008, the World Health Organization (WHO) recognized severe depression as the third leading cause of global disease burden, based on factors including financial costs, mortality, morbidity, and associated health consequences. Projections indicate that by 2030, severe depression is expected to emerge as the leading cause of global disease burden [4].

First-line treatments for MDD, encompassing both psychopharmacological and psychological interventions, do not provide sufficient efficacy for all patients, with approximately one-third remaining unresponsive to these approaches [5]. While a universal definition of treatment-resistant depression (TRD) is lacking, it represents the subset of MDD patients unresponsive to treatment and is typically defined by a failure to achieve a satisfactory clinical response after at least two different antidepressant treatments administered at adequate doses and duration [6]. Studies that explored cost of illness showed that MDD results in significant economic burdens, with TRD responsible for over half of these global costs. TRD is also associated with greater psychosocial impairment, higher disability and absenteeism, increased caregiver strain, and elevated rates of suicidality, including completed suicide [7]. Importantly, TRD presents a unique clinical challenge, as it often requires more complex, multimodal treatment strategies and is linked to poorer long-term outcomes, including elevated suicidality. Managing TRD often requires advanced therapeutic approaches, including augmentation with other medications, ketamine or esketamine infusions, transcranial magnetic stimulation, or specialized psychotherapies [8]. Furthermore, electroconvulsive therapy (ECT) has emerged as a preferred intervention for managing TRD [9], demonstrating both rapid antidepressant effects [10] and a reduction in suicidal ideation [11]. ECT is recognized as an effective treatment for both the acute and maintenance phases of TRD, with early findings indicating that it may be comparably effective to intravenous ketamine during the acute phase [7]. Therefore, recognizing and distinguishing TRD from general MDD is critical for optimizing treatment planning, resource allocation, and research efforts for improving therapeutic options.

ECT is a medical procedure in which precisely controlled electrical currents are administered to the brain under general anesthesia, intentionally inducing a generalized seizure for therapeutic purposes [12]. Since its discovery in the early 20th century, ECT has undergone significant advancements and remains a cornerstone treatment for severe mood disorders, particularly in cases of treatment-resistant MDD [13,14]. The evolution of ECT has led to substantial advancements in anesthesia techniques, electrode placement, and dosage optimization [15]. These improvements have not only enhanced the safety profile of ECT but have also significantly reduced the cognitive side effects that historically contributed to its controversial reputation. In comparison to alternative therapeutic options, ECT is the most effective treatment for symptom remission in MDD patients [16]. Response rates for ECT are notably high, ranging from 60% to 80%, with clinical improvement occurring more rapidly than with standard pharmacological treatments. Therefore, ECT is considered as one of the most potent and swift-acting therapies for affective disorders [17]. Moreover, research indicates that ECT can significantly reduce the duration of hospital stays and decrease the frequency of hospitalizations over a three-year period for patients undergoing maintenance ECT sessions [18]. The efficacy of ECT is strongly supported by robust clinical evidence, consistently showing superior outcomes in managing depression and other mood disorders, including bipolar depression, mania, and certain subtypes of schizophrenia [14,19,20]. However, variations in ECT protocols—electrode placement and stimulus parameters (pulse amplitude, shape, and width, and train frequency, directionality, polarity, and duration)—can influence neurobiological effects, while individualizing these parameters may improve therapeutic response [21]. Furthermore, limitations related to translational potential of animal to human studies, including species differences along with variations in ECT protocols, represent an enormous challenge. Therefore, results obtained from preclinical ECT studies should be taken with cautious interpretation when applied to humans.

The exact mechanism of action of ECT remains unclear, though significant scientific progress has been made in recent years. Several theories have been previously proposed, categorized into neurophysiological, neurobiochemical, and neuroplastic processes, which include effects on neurotransmitters, neurotrophic factors, the immune system, the hypothalamic–pituitary–adrenal (HPA) axis, neuroplasticity, epigenetic changes, brain neurophysiology, circuitry, and structure [22]. Despite extensive clinical and preclinical investigations conducted up to 2025 and its established utilization for over 80 years, the precise molecular mechanisms driving its efficacy remain incompletely understood. Consequently, a deeper comprehension of how ECT operates is essential for illuminating the underlying causes of severe MDD and advancing personalized treatment strategies for these patients. Hence, the aim of our review is to present the most discussed neurobiological mechanisms and associated signaling pathways involved in ECT’s mechanism of action. A comprehensive electronic search was conducted using the following databases: Web of Science, PubMed, and SCOPUS. The search included studies published up to Jun 2025, with no restriction on publication year, but limited to articles published in English. The search strategy combined keywords and medical subject headings (MeSH) relevant to the topic, including terms such as “electroconvulsive therapy”, “depression”, “major depressive disorder”, “treatment-resistant depression”, “neurobiology”, “neurotransmitters”, “neuropeptides”, “neuroplasticity”, “molecular mechanisms”, “oxidative stress”, “apoptosis”, “inflammation”, and “mitochondria”. Boolean operators (AND, OR) were used to refine the search. Additional references were identified through manual screening of the bibliographies of selected articles by three independent researchers (E.F., M.M., and N.M.) to ensure the inclusion of all relevant studies.

## 2. Understanding the Mechanisms of ECT: Key Theories

In previous decades, several theories have been proposed in order to elucidate the precise mechanism underlying the antidepressant effects of ECT. These theories capture the diverse neurobiological alterations induced by ECT and emphasize the various physiological systems that contribute to its therapeutic effects.

### 2.1. Memory Disruption and the Abandoned Amnesia Hypothesis

One of the earliest theories, now largely outdated, was the amnesia hypothesis, which suggested that ECT’s efficacy resulted from disruption of particularly autobiographic memory [23] and memory of emotionally charged or trauma-related events that contributed to symptom onset [24]. Subsequently, this hypothesis led to multiple unsupported ECT administrations per session to enhance amnesia [25,26]. Neuroimaging studies have shown dynamic changes in memory-related brain structures such as the hippocampus during the course of ECT treatment [27]. Early hippocampal volume increases may contribute to cognitive side effects, while later normalization has been associated with cognitive recovery. Also, one study observed increased theta activity in the left medial temporal lobe during the interictal state of bilateral ECT, correlating with transient retrograde amnesia, which suggests functional suppression of memory-related brain regions during ECT treatment [28]. These findings suggest that ECT induces reversible structural and functional changes in brain regions critical to memory [27]. Moreover, the release of endogenous opioids (e.g., beta-endorphin, Met-enkephalin) during ECT has been linked to memory loss, and the administration of naloxone has been shown to reverse these effects [26]. However, this hypothesis was abandoned when research showed that right unilateral or bifrontal placements with ultrabrief pulses caused less amnesia than bitemporal placements while maintaining efficacy [29,30].

### 2.2. The Anticonvulsant Hypothesis

In contrast to the memory-based model, the anticonvulsant hypothesis focuses on neurophysiological inhibition. It emerged from the observation that during ECT, both seizure threshold increases and seizure duration decreases. This led to the hypothesis that the inhibitory brain processes linked to the rising seizure threshold also contribute to depression relief. Supporting evidence from electroencephalogram (EEG) and cerebral blood flow studies shows a suppression of neural activity, particularly in the frontal lobes, after ECT, which correlates with its antidepressant effects [31]. However, later studies have failed to replicate the correlation between an increase in seizure threshold and antidepressant outcomes [32], and magnetic resonance spectroscopy (MRS) has shown no significant gamma-aminobutyric acid (GABA) changes related to ECT’s efficacy [33].

### 2.3. The Neurogenesis Hypothesis

Moving from electrical activity to cellular remodeling, the neurogenesis hypothesis suggests that the therapeutic effects of ECT are driven by an increase in the number of neurons or the strengthening of connections between neurons [34]. The theory is based on neurotrophic effects occurring after electroconvulsive seizures [35], with additional studies reporting amplified signaling of brain-derived growth factor (BDNF) in numerous brain areas and vascular endothelial growth factor (VEGF) in the hippocampus after exposure to electroconvulsive seizures [35], as well as increased precursor cell proliferation in the subgranular zone of the hippocampal dentate gyrus (DG) in the monkey hippocampus [36]. Unlike the anticonvulsant hypothesis, which emphasizes functional suppression, this theory highlights long-term structural adaptation.

### 2.4. The Neuroendocrine Hypothesis

The neuroendocrine hypothesis of ECT adds a hormonal dimension, suggesting that seizures activate the HPA axis, as evidenced by a postictal surge in blood levels of adrenocorticotropic hormone, cortisol, and prolactin [37]. It has been reported that ECT induces a rapid increase in serum concentrations of these hormones, suggesting a significant stimulation of the HPA axis [38]. Additionally, research indicates that ECT decreases serum levels of cortisol, acting as a regulator of HPA axis activity [39]. These findings support the notion that neuroendocrine responses play an important role in the antidepressant efficacy of ECT.

**Figure 1:**
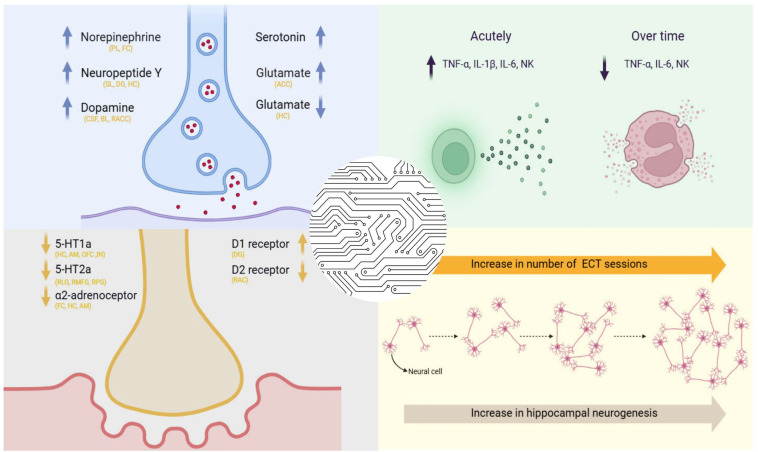
Key theories of potential mechanisms of ECT. The neurotransmitter theory (**top-left**). The cytokine theory (**top-right**). The receptors theory (**bottom-left**). The neurotrophic theory (**bottom-right**).

To date, four main hypotheses have survived in an attempt to explain the potential mechanisms of action of ECT, including the neuroplasticity hypothesis, neurotransmitter hypothesis, receptor hypothesis, and cytokine hypothesis (Figure 1). In Table 1, we summarize both the clinical and preclinical evidence supporting these four hypotheses related to ECT’s mechanisms of action.

### 2.5. The Neuroplasticity Hypothesis

The neuroplasticity (or neurotrophic) hypothesis posits that morphological changes—such as neurogenesis, gliogenesis, or alterations in dendritic or axonal arborization of existing neurons—are critical for the antidepressant effects achieved with ECT [63]. Preclinical animal studies, particularly in rodent models, have demonstrated that electroconvulsive stimulation (ECS) induces a dose-dependent increase in neurogenesis within the DG of the hippocampus [64]. However, it remains unclear to what extent these changes mirror the neuroplastic responses observed in human ECT due to species-specific neurodevelopmental and anatomical differences. These differences in neurodevelopment, brain complexity, and circuit organization between rodents and humans complicate translation. Additionally, ECS protocols in animals often employ stimulation parameters that differ significantly from those used in clinical ECT, limiting validity. Additionally, clinical studies reported increased levels of plasma BDNF in patients with treatment-resistant schizophrenia after ECT [40]. ECT’s beneficial effects can, at least partially, arise from the induction of BDNF production, which, in turn, can affect neuronal proliferation in the DG and the sprouting of its efferent fibers [65].

A growing body of evidence suggests that glutamatergic signaling plays a central role in mediating these neuroplastic changes. Glutamate, as the main excitatory neurotransmitter in the central nervous system, seems to play an important role in regulating mood and is believed to contribute to the therapeutic effects observed with rapid-acting antidepressive treatments. Glutamate acts through receptors like α-amino-3-hydroxy-5-methyl-4-isoxazolepropionic acid (AMPA), N-methyl-D-aspartate (NMDA), and kainite—often functioning together in complex networks—with the NMDA receptor playing a crucial role in synaptic plasticity, long-term potentiation, and memory formation [66]. Rapid-acting antidepressant treatments like ketamine have been shown to modulate glutamate neurotransmission in ways that promote synaptic remodeling. Ketamine, a non-competitive NMDA receptor antagonist, initiates a cascade that begins with NMDAR inhibition on GABAergic interneurons, leading to disinhibition of glutamatergic pyramidal neurons and a surge in glutamate release [67]. This increase in extracellular glutamate subsequently activates postsynaptic AMPA receptors, enhancing synaptic transmission and initiating downstream signaling pathways involving BDNF release and the mammalian target of rapamycin (mTOR) signaling, which are crucial for synaptogenesis [68,69]. Notably, ECT appears to engage similar molecular pathways. Repeated ECT has been shown to upregulate mRNA of AMPA receptor subunits, particularly GluR1, in hippocampal regions such as DG, CA1, and CA3 [70]. This upregulation suggests enhanced AMPA-receptor-mediated synaptic transmission, which is crucial for synaptic plasticity and may underlie the therapeutic effects of ECT. In summary, the evidence suggests that ECS and ketamine share common neuroplastic mechanisms—particularly involving hippocampal neurogenesis, BDNF upregulation, and enhanced glutamatergic function—which may underlie their rapid antidepressant effects and provide a basis for future treatment strategies for patients with severe depression [71]. Nevertheless, some previous studies contradict this view, showing that ECT may even decrease glutamatergic activity in certain regions [72], indicating that the antidepressant effect may depend on restoring homeostatic balance rather than uniformly increasing excitatory transmission. This complexity highlights the need for further research to clarify the region-specific and temporal dynamics of glutamate signaling in response to ECT.

### 2.6. The Neurotransmitter Hypothesis

Another major theory, the neurotransmitter hypothesis, is based on the impact of ECT on monoamine neurotransmitter functioning, such as the enhancement of serotoninergic transmission [73]. Preclinical studies have demonstrated that ECT increases serotonergic neurotransmission, with enhanced expression and activity in the hippocampus and prefrontal cortex (PFC) of both postsynaptic serotonin 1A receptor (5-HT1A) and serotonin 2A receptor (5-HT2A) receptors. In human studies, it has been demonstrated that the binding of both 5-HT1A and 5-HT2A receptors is generally reduced after ECT [52]. Additionally, ECT has been found to affect the GABA system, the primary inhibitory neurotransmitter in the brain, by increasing GABAergic tone and enhancing GABA transmission, thus contributing to its anticonvulsant and anxiolytic effects. Furthermore, the same study showed that ECT-induced activation of the dopamine system likely contributes to the alleviation of depressive and anxious symptoms, accompanied by improvements in motivation, concentration, and attention [74]. Collectively, these findings underscore the multifaceted impact of ECT on neurotransmitter systems, which is central to its efficacy in treating depressive disorders.

### 2.7. The Receptor Hypothesis

Closely related to neurotransmission is the receptor hypothesis, which proposes that an increased affinity of α2 adrenergic receptors is present in the frontal cortex (FC) and hippocampus (CA) in depressive patients [75,76], while this affinity decreases following ECT [76]. At the same time, ECT can influence the expression of genes encoding dopamine receptors, leading to an upregulation of dopamine D1 receptors in the hippocampal CA3 region, which contributes to the treatment of severe mental disorders [50]. These changes suggest a fine-tuning of neuronal sensitivity, refining the effects proposed in the broader neurotransmitter model.

### 2.8. The Cytokine Hypothesis

Finally, the cytokine hypothesis explains that the mechanisms of ECT are related to alterations in cytokine levels after ECT sessions, specifically the levels of interleukin (IL)-6 and tumor necrosis factor-α (TNF-α), while these markers significantly decrease after ECT [77]. This model complements the neuroendocrine hypothesis, as both involve systemic responses outside the central nervous system, and it aligns with the neuroplasticity theory by implicating inflammation in neurodegeneration and plasticity.

While each theory emphasizes different mechanisms—electrical, chemical, structural, hormonal, or immunological—they are not mutually exclusive. Instead, they may reflect different levels of the same therapeutic cascade. For example, neuroendocrine and cytokine changes may create a biochemical environment that promotes neuroplasticity, while neurotransmitter shifts can influence both seizure threshold and structural adaptation. However, further research is needed to fully integrate these mechanisms into a comprehensive understanding of ECT’s efficacy.

## 3. Neurotransmitter and Neuropeptide Modulation by ECT

### 3.1. Modulation of Neurotransmitter Systems Following Electroconvulsive Therapy

Previous research on depression and other psychiatric diseases has focused on exploring the relationship between various neurotransmitter systems and the pathophysiology of these conditions. There is a well-established consensus that at least three neurotransmitter systems—serotonin, noradrenaline, and dopamine—are crucial in the pathogenesis of MDD. This is supported by extensive evidence, including studies utilizing animal models, neuroimaging techniques, genetic analyses, and the pharmacological effects of antidepressant medications, which specifically target one or more components of these neurotransmitter systems. Furthermore, a meta-analysis of monoamine depletion studies has demonstrated an indirect correlation between monoamine levels and mood regulation [78]. In Table 2, we summarize the main changes related to neurotransmitter receptors reported in clinical and preclinical studies following ECT.

ECT modulates the serotonergic system through complex, region-specific receptor changes. Preclinical studies have shown enhanced serotonergic neurotransmission, including upregulation of 5-HT1A and 5-HT2A receptors, though findings vary [89,90]. Some studies report increased 5-HT2A binding without corresponding changes in 5-HT1A mRNA or binding [79,91], while others observed reduced 5-HT2A receptor binding post-ECT, with normalization over time [54]. In MDD patients resistant to antidepressants, ECT has been associated with decreased 5-HT1A receptor binding in emotion-related areas like the amygdala (AM), anterior cingulate cortex (ACC), orbitofrontal cortex (OFC), and insula (IN) [51], though these results are not universally replicated [53]. Reductions in 5-HT2A receptor binding in regions such as the medial frontal and parahippocampal gyri also correlate with symptom improvement [52]. These findings align with studies conducted on non-human primates and research on antidepressant treatments [54,92,93], highlighting the potential role of 5-HT2A receptor modulation as an important mechanism underlying ECT’s therapeutic effects.

In contrast to serotonin, where discrepancies exist between rodent and human studies, research on the effects of ECT on the dopaminergic system has demonstrated a relatively high degree of consistency across both. Post-treatment increases in dopamine metabolites (HVA) and serotonin metabolites (5-HIAA), as well as elevated cerebrospinal fluid NPY-like immunoreactivity (LI), were observed in depressed patients [94]. Responders showed higher baseline HVA levels and a subsequent reduction after five weeks, correlating with HDRS improvement [95]. Receptor-level changes include decreased D2 receptor binding in the rostral ACC [80] and increased D1 receptor expression in the DG [50]. In animal models, ECT led to transient increases in dopamine transporter binding [55] and upregulation of D3 receptor mRNA and binding in the nucleus accumbens shell [81]. Prolactin elevation post-ECT further suggests dopaminergic activation [78]. Genetic studies indicate that dopamine D2 receptor (DRD2) gene C957T (rs6277) and the catechol-O-methyltransferase (COMT) gene Val158Met (rs4680) polymorphisms may influence ECT response [96].

ECT appears to enhance noradrenergic activity primarily through α2-adrenoceptor downregulation in the FC, hippocampus, and AM in preclinical models [48]. While early clinical studies noted increases in plasma norepinephrine post-ECT [83], later findings show mixed results, including reduced NE levels post-treatment without consistent correlation to clinical improvement [84,97]. Epinephrine reduction has been associated with ECT response [46]. It is essential to recognize that monoaminergic systems do not function in isolation but rather interact dynamically. NE modulates dopamine release in the ventral tegmental area (VTA) via α1- and α2-adrenoceptors, while dopamine inhibits NE release from the locus coeruleus. Additionally, both neurotransmitters facilitate serotonin release via α1 (NE) and D2 (dopamine) receptor activation [98].

Glutamate is another neurotransmitter implicated in mood regulation and the therapeutic effects of ECT. Dong and colleagues demonstrated that depressed rats exhibit elevated glutamate levels, which decreased in the hippocampus following ECT [88]. Additionally, an increased glutamate-to-GABA ratio has been observed in the hippocampus and PFC in rodent models of depression [99]. In human studies, alterations in glutamate levels have also been reported. Postmortem analyses of patients with affective disorders revealed increased glutamate concentrations in the FC [100], while reductions were noted in the AM, dorsolateral PFC, and ACC [101]. Notably, ECT has been shown to normalize glutamate concentrations in the ACC in MDD patients, which was in correlation with therapeutic response [86]. Another study reported an increase in glutamate levels in the ACC and a decrease in the hippocampus after ECT in MDD patients [72]. Pfleiderer and colleagues previously demonstrated that ECT induces a significant increase in glutamate levels in the left ACC specifically in responders, whereas non-responders showed no statistically significant change [87], while others have failed to detect significant glutamate alterations following ECT [47]. These discrepancies may be attributed to various factors, including differences in study design, patient populations, timing of measurements post-ECT, and the specific brain regions examined. Moreover, the relationship between glutamate levels and AMPA receptor activation is complex. While increased glutamate can enhance AMPA-receptor-mediated synaptic transmission, excessive glutamatergic activity may lead to excitotoxicity [102]. Therefore, ECT-induced changes in glutamate concentrations may have varying effects on AMPA receptor function, depending on the context and extent of these changes.

ECT exerts its therapeutic effects through complex interactions within serotonergic, dopaminergic, noradrenergic, and glutamatergic systems, leading to neurotransmitter modulation and receptor alterations. Overall, the available evidence underscores the multifaceted neurochemical effects of ECT, highlighting its capacity to restore balance across multiple neurotransmitter systems. While these findings provide valuable insights into the biological underpinnings of ECT, further research is required to fully elucidate its mechanisms of action and optimize its clinical application in MDD and other psychiatric conditions.

### 3.2. Alterations in Neuropeptide Expression Associated with Electroconvulsive Therapy

Neuropeptides, acting as neuromodulators often co-released with neurotransmitters, regulate numerous physiological functions and have been increasingly recognized for their roles in stress adaptation, anxiety, and depression, with expanding research highlighting their potential as targets for novel diagnostics and therapies [103].

NPY, a key regulator of feeding, circadian rhythms, and memory, has been implicated in the etiopathogenesis of MDD [104]. A study examining the effects of antidepressants on NPY reported a significant increase in serum NPY concentration in depressed patients, with the most pronounced elevation observed after six months of treatment [105].

Earlier studies have found that there is an increase in NPY-LI in the right and left hippocampus, occipital cortex, and FC of rats 15 min, 60 min, and 24 h after the last ECS, with a simultaneous increase in the concentration of Neurokinin A in the right and left hippocampus. Concentrations of both these neuropeptides returned to normal 15 days after the last ECS. The same study showed no significant changes in the concentrations of Substance P or Neurotensin compared to the concentrations of these neuropeptides after sham ECS [106]. In some preclinical studies, it was shown that repeated ECS after two or more applications significantly increased the expression of the NPY gene in the hilus of the DG and the piriform cortex, with the largest increase in the 14th cycle of therapy, compared to naive and sham-treated rats. The same study also showed an increase in the level of SS mRNA in the DG, with a maximum after 18 applications of ECS, but to a lesser extent than the level of NPI gene expression [107]. Similarly, Altar and colleagues demonstrated that ECS increases the expression of NPY pathway genes, followed by elevated NPY levels in the hippocampus and DG two weeks post-stimulation [35]. These results were consistent with those obtained in a study by Nikisch and Mathé, who showed that in patients on ECT treatment, there was an increase in the concentration of NPY in the CSF one week after the eighth cycle of ECT, with a decrease in CRH levels [94].

Regarding other neuropeptides, a study by Pedersen and Schou showed that after long-term ECT, there is no change in the binding of titrated enkephalinamides to opioid receptors in membranes in the cerebral cortex, hippocampus, basal ganglia, or the rest of the forebrain [108].

Investigating the effects of ECT on β endorphin levels in nine MDD patients, *Weizman* and colleagues came to the result that there is a significant increase in the level of plasma β endorphins immediately after the first and sixth ECT session compared to the levels before the treatment, as well as 24 h after the 6th session of ECT, while levels 24 h after the first session were not significantly changed compared to the levels before the start of therapy [109].

## 4. The Role of Neuroplasticity, Functional Network Reorganization, and Neuroanatomical Changes in the Therapeutic Effects of ECT

An increasing amount of evidence suggests that neuroplasticity—which refers to the ability of the brain to undergo structural and functional changes in response to different stimuli, including learning, experience, and injury—plays a crucial role in the therapeutic effects of ECT. It consists of changes in synaptic connections, synaptic remodeling, dendritic and axonal remodeling, neurogenesis (particularly in the hippocampus and PFC), and synaptic pruning and thereby enables essential processes such as the acquisition of various skills, the formation of memories, and the recovery of nerve tissue after damage, all of which help our brain to adapt dynamically during our lifetime [64,110,111,112].

ECT induces extensive neuroplastic changes across neocortical, limbic, and paralimbic areas, with these alterations closely linked to the degree of the antidepressant response. In Table 3, we present the most discussed preclinical and clinical investigations related to structural and functional changes in the brain after ECT.

Various studies showed that ECT induced neuroplasticity in the hippocampus and AM, which was associated with improved clinical response and pronounced in regions with prominent connections to the ventromedial PFC and other limbic structures. Both hippocampal and AM volumes increased following ECT and correlated with an evident improvement of symptoms [123,124,125]. A bilateral increase in hippocampal volume has been reported one week after ECT, but these changes were no longer detectable at a six-month follow-up [113]. Also, post-ECT increases in hippocampal and AM gray matter volume did not correlate with improvements in depression or cognitive function in patients receiving right unilateral ECT [114], while some studies did not assess the relationship between these changes and clinical outcomes [115]. While most studies indicate no clear link between hippocampal volume increases and antidepressant efficacy, some research suggests a connection to cognitive impairment [116]. Overall, changes in hippocampal volume and function induced by ECT may indicate neuroplasticity; however, these effects are often temporary and do not consistently correlate with clinical outcomes in depression or cognitive side effects.

While ECT-induced neurobiological changes are widely supported across multiple studies, the literature often presents conflicting findings, including those related to BDNF levels and hippocampal volume. These inconsistencies likely arise from several methodological and biological sources. For example, BDNF levels have been measured in both serum and plasma, at various time points, and across populations with differing medication regimens and clinical characteristics. Some studies measured BDNF immediately post-ECT, while others assessed levels days or weeks later, which may capture different phases of neuroplastic adaptation. Similarly, changes in cytokines such as IL-6 or TNF-α are often transient and may depend on whether measurements were taken acutely or during follow-up. Variability in ECT protocols—such as electrode placement (bitemporal vs. unilateral), number of sessions, and seizure threshold titration—can also influence outcomes. Furthermore, individual differences in patient age, sex, diagnosis (e.g., unipolar vs. bipolar depression), and baseline inflammation or oxidative stress may modify treatment response.

It is important to recognize that the molecular and neurobiological effects of ECT are not uniform across all patients but rather depend heavily on the specific stimulation parameters used. Variations in electrode placement (e.g., right unilateral, bitemporal, bifrontal), pulse width, frequency, current amplitude, and total charge can significantly influence both the clinical response and the nature of neurobiological changes induced by ECT. For instance, bitemporal ECT is associated with more robust hippocampal volume increases but also carries a higher risk of cognitive side effects, while right unilateral ECT may induce subtler structural changes with a more favorable cognitive profile [126]. Interestingly, a preclinical study demonstrated that a brief pulse width versus an ultrabrief pulse width can alter seizure quality and consequently affect antidepressant-related molecular, cellular, and behavioral changes [127]. These protocol-dependent effects likely contribute to the heterogeneous findings observed across studies investigating ECT-induced neurobiological changes. Therefore, future research should systematically consider how specific ECT parameters shape molecular outcomes to better tailor treatment protocols for maximizing efficacy while minimizing side effects.

Beyond the hippocampus, there is a smaller body of research on ECT-induced neuroplasticity in other brain regions and white matter. Volumetric increases have also been observed in the ACC, postcentral gyrus, fusiform gyrus, medial PFC, supplementary motor cortex, IN, and striatum [128]. Moreover, variations in ACC thickness, which can distinguish between treatment responders and non-responders early, may serve as a biomarker for overall clinical outcomes [129]. Lyden and colleagues found increased fractional anisotropy in the bilateral ACC, forceps minor, and left superior longitudinal fasciculus following ECT, which were associated with reductions in depressive symptoms of MDD patients. This suggests that ECT may enhance the integrity of fronto-limbic pathways involved in mood regulation [117].

The neuroplasticity and neurogenesis hypothesis suggests that the therapeutic effects of ECT are driven by an increase in the number of neurons or the strengthening of neural connections [34]. Preclinical research has demonstrated that ECS, the animal model equivalent of ECT, increases the proliferation of neural progenitor cells in the DG of the hippocampus, a region crucial for memory processing and emotional regulation as well as and bromodeoxyuridine (BrdU)-positive cells in the same region [64,119,120]. When extended to adult non-human primates, ECS was found to increase precursor cell proliferation in the subgranular zone of the DG, with most of these cells differentiating into either neurons or endothelial cells [36]. Also, ECT has been shown to modulate synaptic plasticity by increasing the expression of BDNF, a key molecule involved in neuronal survival, synaptic strength, and adaptive responses to stress and VEGF, specifically in the hippocampus [121]. BDNF levels are often reduced in MDD patients, and their restoration following ECT has been associated with symptom improvement [130]. Furthermore, ECT alters the expression of genes and proteins associated with synaptic function, including glutamatergic and gamma-aminobutyric acid (GABA)-ergic signaling, which are critical for maintaining excitatory–inhibitory balance in the brain. Various studies indicate that ECS enhances neurogenesis by increasing the volume of certain brain regions, which correlates with improved behavioral outcomes and neuroplasticity [131,132]. The protein Homer-1, primarily found in two forms—short (Homer1a) and long (Homer1b/c)—is found to be crucial for postsynaptic density, connecting metabotropic glutamate receptors (mGluRs), and regulating their signaling pathways [133]. Homer1a, a rapidly produced variant in response to neuronal activity, competes with the more stable Homer1b/c for mGluR binding. This balance is of particular importance for neuronal plasticity; Homer1a dominance promotes homeostatic plasticity, while Homer1b/c is associated with heightened activation [133,134]. Homer1a, which is mainly located in the CA1 hippocampus, is activated by neuronal stimulation, such as seizure activity [122,133]. It increases Alpha-amino-3-hydroxy-5-methyl-4-isoxazolepropionic acid (AMPA) receptor clustering, enhancing synaptic transmission and excitatory postsynaptic potential (EPSC) without changing presynaptic glutamate release. Additionally, Homer1a modulates the mGluR-IP3 signaling pathway, reducing excitability in pyramidal neurons and acting as a negative feedback mechanism to prevent excessive excitation. Research shows that increased Homer1a in the medial PFC has antidepressant effects, while lower levels are linked to depression [135]. In the hippocampus, high Homer1a may increase stress vulnerability [122]. Homer1 also regulates the HPA axis independently of mGluR1/5. By interacting with mGluR1/5 and NMDA receptors, Homer1a can induce rapid antidepressant responses [136]. Thus, Homer1a is essential for mediating antidepressant effects, with its splice variants, Homer1b/c, having distinct regulatory roles. ECS remodels neuroplasticity by balancing mGluR1/5 and AMPA receptors, leading to rapid antidepressant effects. It activates presynaptic glutamatergic neurons and inhibits GABAergic neurons, resulting in increased glutamate release and AMPA receptor activation while inhibiting NMDA receptors. This process promotes the release of BDNF, which activates the TrkB receptor and subsequently signals Akt to mTORC1, encouraging neurogenesis. Additionally, Homer1 disrupts dysfunctional complexes with mGluR1/5 and partially opens the BK channel, contributing to the hyperpolarization of the postsynaptic neuron and enhancing the antidepressant effect [137].

Additionally, neuroplasticity induced by ECT goes beyond just molecular and cellular changes; it also affects the functional connectivity within large-scale brain networks. Depression is often associated with dysregulation in the default mode network (DMN), which is linked to self-referential thinking and rumination. Functional neuroimaging studies indicate that ECT decreases hyperconnectivity within the DMN while enhancing connectivity in cognitive control networks, such as the central executive network (CEN). These connectivity changes, such as altered communication between the medial and ventrolateral PFC, as well as between the dorsomedial PFC and posterior cingulate cortex, have been associated with clinical improvement and contributes to mood stabilization and cognitive recovery [118,138].

In conclusion, the current neurobiological model explaining the effects of ECT suggests that patients with MDD have reduced neuroplasticity prior to the start of ECT treatment, affecting the brain’s inherent ability to change structurally and functionally in response to external and internal stimuli. It is this impaired neuroplasticity, which is thought to play a key role in MDD and in limiting the brain’s adaptability and recovery mechanisms, that ECT is thought to affect, thereby alleviating the clinical signs of MDD. Each ECT session induces temporary brain disruption, which can cause postictal confusion but also triggers physiological changes like reduced N-acetylaspartate levels, altered connectivity, and changes in white matter integrity. This disruption leads to a heightened state of neuroplasticity, promoting the reorganization of neural circuits related to depression. It has been also suggested that excessive ECT dosing may result in significant structural and functional changes, providing both antidepressant and cognitive side effects. Conversely, insufficient dosing may not yield an adequate antidepressant response but could minimize side effects. Understanding these dynamics can help optimize ECT protocols to balance benefits and risks [34,128].

While preclinical studies using ECS in animals have been invaluable in advancing our understanding of the biological underpinnings of ECT, caution must be exercised when interpreting these findings in the context of human psychiatry. Notably, species-specific differences in brain anatomy, neurochemical pathways, and developmental timelines may substantially alter the effects of ECS. As we already mentioned, ECS protocols used in rodent models often involve higher frequencies, current intensities, and different electrode placements than those used clinically, which may produce effects that are not representative of human ECT. Therefore, although preclinical research provides critical mechanistic insights, these findings should be viewed as hypothesis-generating rather than directly translatable to clinical outcomes in humans.

It is worth mentioning that although ECT is a highly effective treatment for severe depression and TRD, inducing significant neuroplastic changes in the brain, its antidepressant effects are often transient, with relapse rates remaining high after treatment cessation. In fact, approximately 51% of patients relapse within 12 months following successful ECT, with the majority relapsing within the first six months [139]. This paradox may arise from several factors: While ECT induces structural and functional changes, these may not be sufficient to maintain long-term mood stabilization without additional therapeutic support. Moreover, depression is a multifactorial disorder involving chronic stress, inflammation, and dysregulated neurocircuitry [140], which may not be fully addressed by ECT alone. Additionally, the brain’s inherent homeostatic mechanisms could counteract the beneficial effects of ECT over time, gradually restoring pre-treatment neural states. Additionally, some studies suggest that maintenance ECT or adjunctive pharmacotherapy may be required to sustain these therapeutic effects [141,142].

## 5. Molecular Pathways Related to ECT

**Figure 2:**
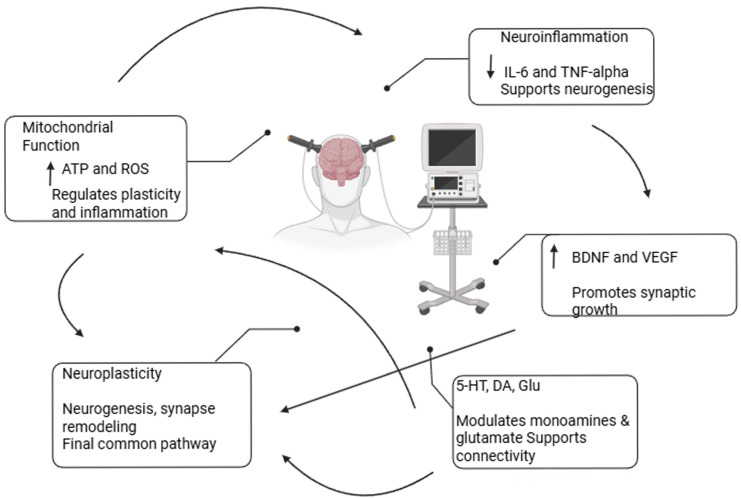
Conceptual integration of neurobiological mechanisms involved in ECT. Mitochondrial activation increases ATP production and modulates ROS levels, initiating signaling cascades that regulate inflammation (e.g., IL-6, TNF-α), promote neurogenesis, and upregulate neurotrophic factors such as BDNF and VEGF. These processes enhance synaptic plasticity and neurotransmitter balance, contributing to therapeutic efficacy in MDD.

Various molecular pathways have been implicated in ECT’s effects, as shown in Figure 2. In the next section, the main molecular mechanisms related to ECT will be explained in detail.

While the previous sections have described the individual mechanisms underlying ECT’s therapeutic effects—such as neurotransmitter modulation, neurotrophic signaling, immune regulation, and oxidative stress—these processes are not isolated. Instead, they are part of a tightly interconnected biological network. In line with emerging evidence, we summarized a synthesized model wherein ECT triggers mitochondrial activation, resulting in elevated ATP production and increased ROS signaling. This mitochondrial response acts as a key upstream modulator of downstream molecular events. ECT induces an acute rise in pro-inflammatory cytokines following early sessions, which subsequently decrease to or below baseline levels after completing the course, suggesting dynamic immune modulation [143]. Concurrently, increases in neurotrophic factors such as BDNF and VEGF have been directly linked to structural plasticity—specifically hippocampal volume growth—highlighting their role in synaptic and dendritic remodeling [144]. These effects converge on enhanced connectivity and restoration of excitatory/inhibitory balance in cortico-limbic networks. Neurotransmitter systems, particularly glutamate, GABA, serotonin, and dopamine, are also modulated in this process, as discussed previously, contributing to improved mood regulation and cognitive function. Therefore, the therapeutic efficacy of ECT likely arises from a systems-level adaptation involving bioenergetics, immune signaling, structural plasticity, and neurotransmission.

Given these complex dynamics, interpreting ECT-induced changes requires careful consideration of methodological and biological variability across studies. In light of the methodological complexities discussed earlier, future studies should adopt standardized protocols for biomarker collection and ECT administration. Stratifying patients based on clinical or biological characteristics may also clarify the context-specific mechanisms of ECT. Therefore, discrepancies across studies should probably be viewed as indicators of context-dependent effects rather than only as inconsistencies undermining ECT’s efficacy.

### 5.1. Neurotrophins and ECT

One of the most extensively studied systems implicated in the pathogenesis of MDD is the neurotrophic system, where it has been shown that antidepressants influence the concentrations of neurotrophins and neurotrophic factors [145]. BDNF is a crucial neurotrophic factor that plays a key role in neuronal development, maintenance, and plasticity in the central nervous system (CNS), particularly by promoting neurogenesis, enhancing synaptic plasticity, and supporting neuronal survival [146]. During ECT, alterations occur in various neurobiochemical molecules, including BDNF but also other neurotrophic factors, further triggering neuroplastic changes within the brain. These modifications, alongside enhanced neuronal proliferation, also have a neuroprotective function. Notably, a single application of ECT has been shown to induce neuronal proliferation in the hippocampal DG of rats, with these new neurons surviving for several months [64,147].

Studies investigating the relationship between baseline BDNF levels and response to drug therapy, as assessed by depression scales, have yielded conflicting results [148,149,150]. Various studies have highlighted a strong association between the response to antidepressant treatment and early increases in serum or plasma BDNF levels during the first few weeks of therapy [58,151,152,153]. Karege and colleagues found that individuals with depression exhibit significantly lower baseline serum BDNF levels, which increase in response to ketamine treatment [154,155]. Also, some studies have shown that patients suffering from TRD have lower BDNF levels before therapy compared to healthy controls and a significant increase in these levels after ECT. It was also found that there is a temporal correlation between clinical response to ECT and increases in BDNF levels, suggesting that BDNF levels could represent a biological marker of remission during ECT sessions [156]. However, some studies which examined the predictive power of baseline BDNF as an indicator of potential response to ECT did not show positive results [130,157,158,159]. Ryan and colleagues assessed baseline BDNF levels in medicated patients with depression and revealed no significant differences in BDNF concentrations between these patients and controls. Additionally, the study showed no notable change in BDNF levels in depressed patients before and after ECT administration [158]. However, some less frequent studies, such as the one conducted by Sorri and colleagues, reported a decrease in BDNF levels during ECT. These contrasting findings have led some researchers to question the reliability of BDNF as a determinant of ECT efficacy [130,158,160,161].

The discrepancies in BDNF levels observed in studies involving depression and ECT may arise due to several factors. First, methodological differences between studies, such as variations in sample size, measurement techniques (e.g., serum vs. plasma BDNF levels), and the timing of BDNF measurements (e.g., pre- and post-treatment time points), could contribute to conflicting results. Sample types can yield varying concentrations of BDNF due to differences in the release and stability of BDNF in different blood compartments. For instance, Sorri and colleagues reported that serum BDNF levels were not affected by ECT, while BDNF plasma levels decreased during the fifth ECT session [130]. This discrepancy underscores the importance of standardizing measurement techniques to ensure comparability across studies. More importantly, variability in BDNF findings post-ECT may also result from poor correlation between central and peripheral BDNF levels and their distinct temporal dynamics, as brain, CSF, and serum BDNF show region-specific and time-dependent expression patterns following ECT [130]. Also, the timing of BDNF measurements relative to ECT sessions can significantly influence observed levels. BDNF levels may peak shortly after treatment and then decline over time. For example, in a study by Mikoteit and colleagues, serum BDNF levels were measured at baseline and at 1, 2, and 6 weeks after ECT. Although baseline measures of low serum BDNF correlated with low HDRS scores, the BDNF levels were not predictive of ECT outcomes [149]. This suggests that the timing of BDNF assessments is critical in understanding the relationship between BDNF levels and treatment response. Second, variations in treatment protocols can differ in terms of the type, dosage, and frequency of antidepressant medications or ECT, which could lead to varying effects on BDNF expression. Some treatments might stimulate BDNF production more robustly than others, while some may not induce noticeable changes in BDNF levels at all. Also, other methodological issues can explain the mixed results, such as heterogeneity between the studies in aspects such disease severity, refractoriness, and concomitant use of pharmacotherapy. Finally, individual variability and genetic differences in patients, particularly variations in genes associated with BDNF production or its receptor or in the pathophysiology of depression, can influence how BDNF levels are regulated in different patients. Depression is a heterogeneous condition, meaning that varying biological mechanisms may contribute to its development in different individuals, which could lead to inconsistent BDNF responses across studies. Overall, in a recent review by Zelada and colleagues, the authors reported lower BDNF serum levels in patients with MDD compared with those in healthy controls, and pharmacological treatments usually led to an increase in these levels, which correlated with an improvement in the clinical presentation. However, there is more controversy in the literature regarding non-pharmacological treatments and BDNF levels in this population of patients [162]. One preclinical study demonstrates that promoter-I-derived BDNF is essential for structural plasticity in BDNF exon 1-expressing neurons of the piriform cortex following ECS [43]. These findings highlight the importance of promoter-specific BDNF regulation in mediating the neuroplastic effects of ECS, offering insight into the molecular mechanisms that may underlie the therapeutic effects of ECT in depression. However, not all studies look at these specific BDNF forms, which may explain why results about ECT’s effects on BDNF are sometimes inconsistent. Notably, while animal studies consistently demonstrate increases in hippocampal BDNF after ECS [163,164], translating these findings to humans remains difficult due to methodological limitations and ethical constraints in measuring central BDNF directly. Moreover, species-specific differences in BDNF gene regulation, receptor distribution, and neuroanatomy may further limit the direct applicability of these findings. The stimulation frequency, electrode positioning, and current intensity used in animal ECS often do not reflect clinical ECT practice, adding another layer of complexity when attempting to infer human outcomes.

Another growth factor hypothesized to play a role in the pathogenesis of MDD is VEGF. VEGF promotes vasculogenesis, angiogenesis, and neurogenesis in the hippocampus, with hypoxia increasing its expression in both the hippocampus and peripheral organs [165]. VEGF also regulates glutamatergic synaptic function, suggesting its role in the pathophysiology of psychiatric disorders [165]. However, studies on VEGF levels in MDD patients have yielded mixed results compared to controls [166]. Minelli and colleagues found that VEGF levels increased in patients undergoing ECT, with a temporal correlation between VEGF levels and symptom improvement, supporting its potential role in ECT’s mechanism of action [167]. In another study which enrolled 67 MDD patients, the authors observed a significant correlation between reduced depressive symptoms and VEGF levels before ECT, suggesting that pre-treatment VEGF levels may predict treatment response [168].

The role of neurotrophic factors, such as nerve growth factor (NGF), neurotrophin-3 (NT3), neurotrophin-3 (NT4), glial-cell-derived neurotrophic factor (GDNF), NPY, and their receptors (TrkA, TrkB, TrkC, p75), in relation to ECT therapy remains insufficiently explored. *Grønli* and colleagues studied NT3, NGF, and NPY levels in patients with affective disorders before and after ECT, finding no significant change in NT3 and NGF but a notable increase in NPY levels [169]. However, the study had limitations, including a small sample size and broader diagnostic criteria, and the authors warranted further research to confirm these findings. Zhang and colleagues found that patients with TRD who responded to ECT (based on a >50% reduction in HDRS scores) had a significant increase in serum GDNF, while non-responders did not [170]. Another study showed decreased GDNF levels in the hippocampus and striatum of rats treated with ECT but increased NGF in the FC and BDNF in the hippocampus, FC, and striatum [171].

There is a lack of studies examining other molecules in the BDNF signaling pathway and the impact of ECT on BDNF and neurotrophic factor receptors. Enomoto and colleagues found that ECT treatment in rats for 10 days led to downregulation of full-length TrkB receptors, potentially in response to elevated mBDNF concentrations, but also an increase in phosphorylated TrkB expression in the dorsal and ventral hippocampus, suggesting enhanced BDNF/TrkB signaling [172]. Schurgers and colleagues found that ECT significantly increased the concentration of molecules involved in the BDNF/TrkB signaling cascade, which negatively correlated with depression scores in TRD patients [42].

Collectively, while several studies have explored the role of neurotrophic factors and their receptors in relation to ECT therapy, the findings remain mixed, and it is hard to bring strong conclusions. Some research suggests that ECT may influence the expression of neurotrophic factors like NPY, GDNF, and BDNF, which could be linked to therapeutic outcomes in patients with TRD. However, the limited sample sizes, varied diagnostic criteria, and insufficient exploration of other molecules in the BDNF signaling cascade indicate that more comprehensive studies are needed to fully understand the underlying mechanisms. Ultimately, future research should aim to clarify the precise role of these factors and their receptors in ECT’s therapeutic effects, potentially offering valuable insights for optimizing depression treatment strategies.

### 5.2. ECT and Immunological Alterations

The involvement of immune system cells in the pathogenesis of depressive and other psychiatric disorders has gained increasing attention in recent years. In this context, it has been hypothesized that ECT may exert its effects, at least in part, through modulation of the immune system, particularly cytokines. It has been shown that in MDD patients, the release of cytokines in response to external stress is enhanced both peripherally and centrally [173], with pro-inflammatory cytokines such as TNF-α, C-reactive protein (CRP), and IL-6 being particularly significant and often recorded in increased levels [173,174]. Previous studies have demonstrated that modulating the activity of the innate immune system by antagonizing the action of certain cytokines can improve depressive symptoms in patients with inflammatory diseases, even without treating the underlying disease. Furthermore, studies in mice have shown that knocking-out genes for TNF-α receptors resulted in decreased anxiety during infections and the development of an antidepressant-like phenotype [175,176,177].

Microglia, the resident macrophages of the CNS, originate from the mesoderm and migrate to the CNS during development, where they play a crucial role in maintaining homeostasis and protecting against injury [178]. They support neuronal survival, differentiation, and circuit formation through BDNF signaling and are involved in synaptic formation related to learning. However, persistently activated microglia can contribute to neurodegenerative diseases by producing high levels of proinflammatory cytokines and chemokines, leading to neuronal dysfunction [178]. In animal models of depression, microglia were shown to be activated, and modulating their activity led to an improvement in the clinical signs of the disease [179]. It has been shown that electrical neuronal stimulation regulates microglia and controls their activation in response to immune challenges. Although electroconvulsive seizures did not affect resting microglia, they induced transcriptomic changes in the retinoic acid receptor α response pathway, which modulated microglial response to immune stimulation [60]. Further research related to exploring the effects of ECS on brain microglia, as well as on astrocytes, would be crucial, as neuronal excitation and glutamate release are known to induce calcium waves and gliotransmission, with ECS promoting increased glial fibrillary acidic protein (GFAP) expression and astrocyte proliferation [60].

Research investigating cytokine levels during ECT treatment has shown that the concentrations of pro-inflammatory cytokines such as TNF-α, IL-1β, and IL-6 increase, particularly in the first hour, following the current application. These early changes in cytokine levels remained consistent throughout the course of therapy, regardless of whether it was an initial or later ECT session, with no reduction in this acute effect through repeated treatments [57,180]. However, Hestad and colleagues observed that after multiple ECT sessions, there was a gradual reduction in TNF-α levels, most notably twenty-four hours and one week after the final treatment. This decrease was not observed in control patients receiving only pharmacotherapy [181]. Additionally, a study by Järventausta and colleagues found that IL-6 levels decreased towards the end of ECT treatment, which correlated with patient response scores, as measured by the Montgomery–Åsberg Depression Rating Scale (MADRS) [59]. An early increase in pro-inflammatory cytokines, such as TNF-α, IL-1β, and IL-6, shortly after ECT sessions (e.g., 3 or 6 h after) was observed by Lehtimäki and colleagues [57], which likely reflects an acute stress or inflammatory response to the electrical stimulation. This is a typical immune activation seen with any acute physiological challenge. However, later measurements, as reported previously [59,181], probably capture longer-term adaptations, during which inflammation may subside as part of a homeostatic or anti-inflammatory rebound mechanism. Concretely, repeated ECT sessions may induce neuroimmune adaptation, gradually dampening the pro-inflammatory response. This aligns with findings that TNF-α and IL-6 levels begin to decrease later in the treatment course, particularly in patients who respond clinically. These changes might indicate a therapeutically relevant immunomodulatory effect, as seen with the IL-6 reductions that correlated with symptom improvement [59].

Kranaster and colleagues investigated the effect of ECT on the activity of innate immune system cells and concluded that ECT likely exerts its therapeutic effects, at least in part, through changes in neuroinflammation. Their study showed a reduction in the concentration of macrophage migration inhibitory factor (MIF) in the cerebrospinal fluid of depressed patients, which is a pro-inflammatory protein implicated in the regulation of the innate immune system and neurogenesis. Additionally, there was a reduction in the serum concentration of the CD14 molecule [182]. Fluitman and colleagues also noted increased concentrations of TNF-α and IL-6 along with an increase in IL-10 production by monocytes after lipopolysaccharide stimulation and a decrease in IFN-γ production by T cells following CD2/CD28 stimulation after ECT application. They further observed a temporary increase in leukocytes, granulocytes, natural killer (NK) cells, and monocytes immediately after ECT, with these levels returning to baseline approximately 30 min post-treatment. Additionaly, repeated ECT sessions showed no cumulative effects on these acute changes. However, sample size and patient heterogeneity prevent clear correlations between these immune changes and depression symptom severity [183]. Similarly, the study by Kronfol and colleagues showed an increase in NK cells immediately after ECT application, with this effect observed in repeated sessions, though long-term effects on NK cells were not monitored [184]. Long-term studies of ECT’s impact on immune cells have indicated that after multiple sessions, certain NK cell subtypes, particularly CD56dimCD16+ cells (which are responsible for cytotoxic activity), decrease in number, while CD56highCD16−/dim cells increase. This shift suggests a reduction in NK cell cytotoxicity, although opposite results are observed within the first 15 min post-treatment. Notably, a correlation was found between the ratio of these NK cell subtypes before ECT and the treatment outcomes [185]. S100B, a calcium-binding protein produced by astrocytes, plays a key role in protecting neurons from oxidative stress, promoting neuron growth, and regulating cell motility, proliferation, and metabolism [186,187,188]. Arts and colleagues studied the concentration of S100B following ECT and found that its serum levels increased 1 and 3 h post-treatment. However, similar to some other studies, they observed no long-term changes in S100B levels after multiple ECT sessions [189].

Emerging evidence suggests that immune system modulation, particularly through cytokine changes, plays an important role in the therapeutic effects of ECT in patients with depression. Despite these findings, significant gaps remain in understanding the precise mechanisms through which ECT interacts with the immune system, and further research is necessary to explore these connections more thoroughly. Inconsistencies in cytokine responses to ECT are influenced by several methodological and biological factors. Variations in study design, such as differences in cytokine measurement timing, sample collection methods, and assay techniques, can lead to significant discrepancies in findings. For instance, cytokine levels fluctuate rapidly after ECT, and the timing of blood sampling can impact the observed concentrations. Additionally, factors like sample handling, storage conditions, and the use of different assay kits can introduce variability in results. Furthermore, patient heterogeneity, including differences in age, sex, medication status, and baseline inflammatory profiles, can contribute to variability in cytokine responses to ECT. These methodological and biological challenges underscore the need for standardized protocols and larger, more homogeneous study populations to better understand the immunological effects of ECT [190]. Also, future studies could explore how ECT modulates immune cell signaling pathways, cytokine receptor expression, and intracellular processes within immune cells. Specifically, how ECT influences the balance between pro-inflammatory and anti-inflammatory responses could provide insight into its therapeutic effects.

### 5.3. Mitochondrial Function and Energy Metabolism During ECT

Mitochondria are dynamic organelles that create an interconnected network within the cytosol. Their morphology is regulated by the processes of fusion and fission, both of which are essential for maintaining optimal mitochondrial function. Fission, mediated by the dynamin-1-like protein (Drp1), contributes significantly to quality control, while fusion, facilitated by mitofusin 1 (Mfn1), mitofusin 2 (Mfn2), and optic atrophy-1 (OPA1), promotes the exchange of mitochondrial components, including proteins, lipids, metabolites, and mitochondrial DNA (mtDNA) [191]. Mitochondria are vital for adenosine triphosphate (ATP) production via the electron transport chain and ATP synthase. The electron transport chain, consisting of complexes I, II, III, and IV in the inner mitochondrial membrane (IMM), creates a proton gradient used by ATP synthase. This process, known as oxidative phosphorylation (OXPHOS), generates reactive oxygen species (ROS) as byproducts [192]. While ROS can act as signaling molecules, excessive amounts may cause protein and lipid oxidation, triggering autophagy, apoptosis, necrosis, and inflammation [193]. Mitochondria produce ATP for Na+-K+-ATPase, crucial for maintaining neuronal membrane potential and regulating Ca^2+^ during synaptic transmission [194].

Mitochondrial dysfunction can lead to neurodegenerative and neuropsychiatric disorders [191], and the relationship between depression and mitochondrial dysfunction has been already established. Initially, depression was supposed to be among the first symptoms of mitochondrial diseases or mutations of mitochondrial or mitochondrion-related genes associated with MDD [195]. Previous research suggests that abnormalities in mitochondrial morphology and function are deeply associated with neuronal function and mood disorders [196]. In a study by Gebara and colleagues, highly anxious rats had more severe depression-like behavior, along with a larger mitochondria area and mitochondria tissue coverage and a higher number of mitochondria–mitochondria contacts in the medium spiny neurons from the nucleus accumbens [197]. Wu and colleagues demonstrated that prenatal exposure to dexamethasone leads to depression-like behavior and mitochondrial damage in the hippocampus [198]. Furthermore, in an animal model of depression, depressive-like symptoms in mice were accompanied with reduced mitochondrial respiratory rates and a dissipated mitochondrial membrane potential in the hippocampus, cortex, and hypothalamus [199]. This suggests that depression may be associated with a disruption in brain energy metabolism due to mitochondrial genetic vulnerability and environmental influence [200]. One recent meta-analysis reported higher mtDNA concentration in circulating blood samples and skin fibroblasts in depressive patients in comparison to healthy individuals, suggesting a potential association between depression and the amount of mtDNA [201]. In patients with confirmed mitochnodrial diseases due to mitochondrial gene mutations, the prevalence of depression was estimated to be 54% [202]. More than 20 years ago, Gardner and colleagues reported that 68% of depressive patients have mtDNA deletions, in comparison to 36% of non-depressive individuals [203], which could be at least partially explained by the activation of inflammatory processes resulting from damaged mtDNA [204,205]. Cases of MDD exhibited rare homoplasmic mutations that may have functional implications in the ATP synthase 8 (ATP8), ATP synthase 6 (ATP6), ND5, and cytochrome b (CYTB) genes. Additionally, patients with depression displayed a subthreshold heteroplasmy rate at a variant located in the displacement loop (D-loop) region of mtDNA [206].

ECS in animal models has been associated with changes in mitochondrial morphology, including fission and fusion dynamics, highlighting the intricate effects of ECT on mitochondrial regulation [207]. Collectively, these findings suggest that ECT may modulate brain metabolism through its effects on mitochondrial enzymes [208]. The interplay between ECT and mitochondrial function is complex and not yet fully elucidated. A deeper understanding of how ECT influences mitochondrial function could lead to optimized treatment protocols and the development of novel therapeutic strategies targeting mitochondrial pathways in patients with mood disorders.

### 5.4. Oxidative Stress and ECT

The brain, which accounts for more than 20% of the total oxygen consumption in the body, relies on oxygen for neuronal function and survival. However, while oxygen is essential for cellular metabolism, its byproducts, including ROS and reactive nitrogen species (RNS), can exert neurotoxic effects when produced in excess [209]. Oxidative stress (OS) is defined as an imbalance between the generation of ROS/RNS and the capacity of the antioxidant defense system, leading to cellular damage, particularly in proteins, lipids, and DNA. Although OS plays a crucial role in maintaining physiological homeostasis, disruptions in redox signaling have been implicated in the onset and progression of various disorders [210]. Excessive ROS accumulation can interfere with neuronal signaling, disrupt cellular integrity, and impair brain function [211]. Furthermore, oxidative injury exposes molecular patterns known as danger-associated molecular patterns (DAMPs), which activate innate immune responses and sterile inflammation in the brain. This inflammatory cascade amplifies the production of proinflammatory cytokines, linking OS and neuroinflammation to the pathophysiology of depression [211].

The brain is particularly vulnerable to OS due to its high metabolic demand, the presence of highly peroxidizable substrates, and relatively low levels of endogenous antioxidants [212]. Increasing evidence suggests that neuroinflammation and OS, known for their roles in neurodegenerative diseases and aging, also contribute to the development of MDD, which is characterized by its multifactorial nature, neuroprogressive aspects, accelerated cellular aging, and a heightened risk of age-related illnesses [213]. Elevated levels of OS biomarkers, such as 8-hydroxydeoxyguanosine and malondialdehyde—a byproduct of the peroxidation of polyunsaturated fatty acids and arachidonic acid—indicate oxidative DNA damage [214]. These biomarkers, together with a reduction in antioxidant enzyme activities, are commonly observed in MDD patients [214]. The “OS hypothesis of depressive disorders” posits that excessive ROS generation and the depletion of antioxidant defenses contribute to structural alterations in the brain [215]. A review by Ait Tayeb and colleagues demonstrated that increased plasma hydrogen peroxide (H_2_O_2_) levels were associated with MDD, while nitric oxide (NO) concentrations showed more variability, with both elevated serum levels and decreased erythrocyte levels observed in MDD patients [216]. Additionally, studies investigating superoxide dismutase (SOD) expression and its activity in serum, plasma, and erythrocytes have yielded inconsistent results, with some studies reporting increases in SOD activity and others showing reductions or no significant differences [216]. One recent meta-analysis indicated increased catalase (CAT) activity in MDD patients [217], which may reflect a compensatory mechanism to mitigate ROS accumulation [216].

Inconsistent results have also been reported regarding lipid oxidative damage, with some studies identifying increased lipid peroxidation in the serum and erythrocytes of MDD patients, while others found no significant differences [218]. Nevertheless, recent research by Bader and colleagues has highlighted the potential of integrating OS biomarkers with clinical and sociodemographic features to improve depression detection and severity assessment using machine learning techniques [219]. This underscores the importance of OS in understanding the pathophysiology of MDD and its potential as a reliable biomarker for personalized treatment strategies.

Alterations in redox balance are increasingly recognized as central to neuroplasticity and neuronal health, which are thought to underlie the therapeutic effects of ECT (REF). The impact of ECT on OS markers has been explored in both human and animal models, though findings remain variable [218,220,221,222]. A systematic review of 11 human studies and 9 animal studies found inconsistent results regarding the influence of ECT on OS markers in circulating blood samples, suggesting that no clear association exists between ECT and OS in psychiatric disorders [218]. For instance, Şahin and colleagues reported lower total antioxidant levels in MDD patients prior to ECT, with a significant increase in antioxidant levels following ECT treatment [221]. Some studies suggest that while ECT may reduce nitrosative stress, it could concurrently induce oxidative DNA damage, highlighting the complex interplay between oxidative and nitrosative stress in ECT’s mechanisms of action [223].

Research in rodent models has produced mixed findings as well. Barichello and colleagues reported a decrease in oxidative damage markers, such as thiobarbituric-acid-reactive substances (TBARS) and protein carbonyls, in the hippocampus following single or repeated electroconvulsive shock (ECS) [220]. The same group also observed reduced oxidative damage in the hippocampus, striatum, and cerebellum but an increase in oxidative damage in the cortex after ECS [224]. Conversely, Župan and colleagues reported increases in hippocampal and cerebellar SOD and glutathione peroxidase (GPX) activities following single ECS-induced seizures [225]. However, other studies demonstrated decreases in SOD and GPX activity across various brain regions, with the reduction in antioxidant enzyme activity persisting for up to 48 h post-stimulation [226]. More recently, a decrease in mitochondrial respiration and an increase in RNA oxidation were observed in rat brain tissue after chronic ECS [227], suggesting that this treatment induces increased OS, which may drive both therapeutic and potentially neurotoxic effects of ECT.

The variability in these findings highlights the complexity of the relationship between ECT and OS. The variability in findings can be attributed to several factors. Firstly, differences in experimental designs, such as variations in ECT protocols, animal models, and human subject characteristics, can lead to divergent results. Secondly, the methodologies used to assess OS markers vary widely, including differences in the types of biomarkers measured (e.g., lipid peroxidation products, antioxidant enzyme activities, DNA/RNA oxidation markers), the timing of sample collection, and the techniques employed for analysis. For instance, studies have reported inconsistent results regarding lipid peroxidation markers, with some identifying increased levels and others finding no significant differences. Similarly, antioxidant enzyme activities, such as those of SOD and catalase, have shown variable responses to ECT. These discrepancies underscore the need for standardized methodologies in future research. Furthermore, the choice of biomarkers and the timing of their measurement are critical factors influencing the outcomes. Some studies have focused on specific time points post-ECT, while others have assessed chronic effects, leading to varying interpretations of ECT’s impact on OS. Additionally, the use of different biomarkers to assess oxidative damage and antioxidant capacity can yield conflicting results, as each marker may reflect distinct aspects of the redox state.

In conclusion, the inconsistencies in the literature regarding the effects of ECT on oxidative stress can be attributed to methodological limitations, including variations in experimental designs, assessment techniques, and biomarker selection. Addressing these issues through standardized protocols and comprehensive analyses is essential for elucidating the role of oxidative stress in ECT’s therapeutic effects and its potential as a biomarker for treatment outcomes. Understanding this relationship may offer critical insights into the mechanisms underlying ECT’s therapeutic effects and inform strategies to mitigate OS-related side effects.

### 5.5. Apoptosis and ECT

Apoptosis is a highly regulated form of programmed cell death that plays a crucial role in development, cellular homeostasis, and the response to various forms of cellular stress. Unlike most other organs, the nervous system exhibits limited neuronal cell division and proliferation following embryonic development. During early stages, an overproduction of neural precursor cells (NPCs) occurs, and excess cells are subsequently eliminated through apoptosis, which is essential for refining neural connectivity and establishing proper brain function. As the nervous system matures, apoptosis thresholds increase significantly, reducing the rate of neuronal cell death and promoting the long-term survival of neurons within a stable, fully integrated neural network [228].

Disruption of apoptotic regulation is associated with various neurodegenerative diseases, such as Alzheimer’s disease, Parkinson’s disease, and Huntington’s disease [229], as well as psychiatric disorders, including depression [230]. Kondratyev and colleagues previously reported that exposure to minimal ECS markedly reduced vulnerability to the neuronal cell death triggered by status epilepticus in rats due to reduced internucleosomal DNA fragmentation and a decrease in apoptosis-like neuronal morphology in the hippocampus and rhinal cortex [231]. On contrary, Zarubenko and colleagues demonstrated a significant level of neuron death in particular parts of the mouse hippocampus following ECS. However, the authors could not interpret whether the neuronal loss occurs due to apoptotic or necrotic processes [232].

One clinical study investigated the levels of serum biomarkers of neuronal injury and astrocytic reactivity in patients with major depressive episode that have undergone acute ECT [233]. Although authors confirmed a temporary increase in serum GFAP, suggesting astrocytic reactivity, no evidence of neuronal injury was observed, while biomarkers such as NfL and t-tau remained unchanged during ECT [233]. On the other hand, ECT was not associated with alterations in E2F transcription factors, a group of proteins involved in different cell functions including apoptosis and cell proliferation [234]. Although E2F1 mRNA levels were significantly lower in peripheral blood of depressed patients in comparison to healthy individuals, ECT did not affect the baseline values [234]. *Ito* and colleagues investigated the effects of different numbers of ECS (1, 10, or 20 applications) on cell proliferation and apoptosis in the subgranular zone of the DG [147]. While the application of 1 or 10 ECSs increased cell proliferation in the observed region, no difference in cell proliferation was observed after 20 ECSs in comparison to control animals [147]. One of the proposed mechanisms for potential antiapoptotic actions of repeated ECT could be related to c-Myc downregulation via ubiquitination–proteasomal degradation and Bad inactivation in the rat FC [235].

## 6. Future Directions

From a molecular perspective, ECT exerts profound and multifaceted effects on the brain, modulating key neurobiological systems, such as neurotransmitter regulation, synaptic plasticity, neurogenesis, inflammation, oxidative stress, and apoptosis. These changes contribute to the therapeutic effects of ECT, particularly in mood disorders like MDD, by promoting neuronal survival, enhancing synaptic connectivity, and fostering neuroplasticity. Although the precise mechanisms remain to be fully elucidated, accumulating scientific evidence strongly supports the notion that ECT induces a coordinated molecular response that not only restores neurochemical balance but also fosters neural regeneration and reorganization, thereby alleviating psychiatric symptoms. Despite this progress, key knowledge gaps remain that must be addressed to improve mechanistic understanding and clinical application.

A major priority is the identification and validation of predictive biomarkers for ECT response and side effects. Future studies should focus on serial measurements of plasma BDNF, inflammatory cytokines (e.g., IL-6, TNF-α), extracellular vesicle markers (e.g., DCX/CD81 ratio), and cortisol dynamics at standardized time points (e.g., baseline, 24 h post-ECT, mid-treatment, post-treatment) during treatment. These markers should be correlated with both clinical outcomes and imaging markers (e.g., hippocampal volume changes). Additionally, multi-modal biomarker panels incorporating peripheral markers, neuroimaging, and genetic variants (e.g., BDNF Val66Met, TrkB polymorphisms) could enable individualized treatment planning and outcome prediction.

The influence of ECT parameters on neurobiological outcomes remains unexplored. Comparative studies—both clinical and preclinical—should systematically assess right unilateral vs. bitemporal and brief vs. ultrabrief pulse protocols. These investigations should evaluate differences in hippocampal neurogenesis and volume (e.g., BrdU+ cell counts), volume change (MRI), cognitive outcomes, and gene expression (e.g., *Homer1a*, *BDNF*, *VEGF*, *CREB*, oxidative stress markers). Stratifying findings by protocol and electrode configuration may clarify discrepancies in treatment outcomes and biomarker profiles observed in the current literature.

Given the transience of hippocampal volume increases and their inconsistent relationship with clinical remission, longitudinal studies are needed. These studies should assess structural and functional brain changes (e.g., hippocampus, amygdala, ACC, DMN, CEN networks) using MRI and resting-state fMRI over at least 6–12 months. Incorporating follow-up neurochemical or genomic markers (e.g., mBDNF, miRNAs) could help determine whether the observed neuroplasticity is durable or compensatory.

To strengthen translational validity, it is essential to standardize ECS protocols in preclinical models. This includes aligning ECS parameters with human ECT, using age- and sex-matched animals, and implementing behavioral endpoints relevant to depression. Importantly, peripheral and central biomarkers (e.g., serum vs. hippocampal BDNF) should be measured in parallel. Broader adoption of harmonized ECS protocols would facilitate cross-study comparison and enhance the predictive value of animal research.

Finally, ECT research must transition from pathway-specific findings toward integrated mechanistic models. Future work should investigate how mitochondrial activity (ATP/ROS balance) intersects with neuroinflammation, glutamatergic plasticity, and BDNF-TrkB signaling and how these collectively drive antidepressant effects. For instance, elucidating how oxidative stress markers (e.g., SOD, catalase, MDA) mediate seizure-induced metabolic demand and subsequent neural recovery could identify new augmentation targets. These models should be tested using systems biology approaches, combining transcriptomic, proteomic, and metabolomic data in time-resolved clinical and animal studies.

By addressing these focused priorities, future research can clarify the mechanisms underlying ECT, resolve current contradictions (e.g., in BDNF or hippocampal volume findings), and enable the development of mechanistically informed, personalized ECT strategies for severe psychiatric illness.

## Figures and Tables

**Table 1:** Summary of clinical and preclinical evidence supporting neurobiological mechanisms of ECT.

Hypothesis	Evidence Type	Population/Model	ECT Characteristics	Main Findings	Ref.
**Neuroplasticity/Neurotrophic**	Clinical	TRS patients (ECT or antipschycotics)	* 4–10 ECT sessions over 4 weeks * bitemporal electrode placement Parameters: pulse width 0.5 ms, frequency 80 Hz, duration 1 s, current 800 mA (individually adjusted and gradually increased)	Post-ECT: * ↑ plasma BDNF levels along with clinical improvement	[40]
	Clinical	TRD patients and HC	* 3 times per week, 12 ECT sessions * bitemporal electrode placement * stimulus dose was initially set at 50 mC, titrated upward until ≥15 s seizure achieved * stimulation dose for subsequent sessions: 1.5 × seizure threshold	Baseline: * ↓ plasma BDNF levels and the ratio of DCX to CD81 in NDEVs in TRD group * ↑ levels of CD81 in the TRD group Post-ECT: *↑ plasma BDNF levels and the ratio of DCX to CD81 in NDEVs * ↔ CD81 in NDEVs	[41]
	Clinical	TRD patients	* twice a week, 6.1 (range 3–11) ECT in total * bitemporal electrode placement * stimulus dose was set at 350 mC and further adjusted according to EEG characteristics and seizure duration	During ECT period: * ↑ mRNA expression of BDNF and ERK1 * ↔ mRNA expression of CREB Follow-up: * ↔mRNA expression of BDNF, ERK1 and CREB ** ↔ mRNA expression of AKT1, NR3C1 and IGF1 during ECT period and follow-up ** ↑ plasma BDNF levels during study	[42]
	Preclinical	Bdnf-e1 −/+ and Bdnf-e1 −/− mice receiving sham or ECS	* 7 ECT sessions during 15 days Parameters: pulse width 3 ms, frequency 100 pulse/s, duration 1 s, current 50 mA	48 h after the final ECS: * ↓ in spine density in piriform cortex neurons in Bdnf-e1 −/− mice * ↔ spine lenght * ↓ % of smaller spines in Bdnf-e1 −/−	[43]
	Preclinical	C57BL/6 male wildtype (WT) and Egr3−/− littermate mice	* ECS once daily during 10 consecutive days * ocular electrode placement Parameters: pulse width 0.3 ms, frequency 260 Hz, duration 100 ms, current 80 mA	11 days after the final ECS: * ↔ dendritic intersections in dorsal/ventral DG of WT and Egr3−/− mice, ↓ dendritic intersections in dorsal DG of Egr3−/− vs. WT, ↑ proximal and ↓ distal dendritic intersections in ventral DG of Egr3−/− vs. WT * ↑ dendritic branches in hippocampus (significant in ventral), ↑ dendritic spines in dorsal and ventral DG (all layers) * ↑ BrdU+ cells in all subregions of dorsal and ventral DG ** serial ECS doubles proliferating cells in dorsal/ventral hippocampus, independent of Egr3	[44]
	Preclinical	Adolescent and adult Sprague–Dawley rats, males and females	* ECS treatment started 4 days after the final BrdU injection * applied once daily during 5 days * earclip electrode placement Baseline parameters: pulse width 0.6 ms, frequency 100 Hz, duration 0.6 ms, current 95 mA	* ↑ BrdU+ cells in adolescent females (1-day) and adult males (all time points); transient in adult females; no effect in adolescent males * ↑ Ki-67^+^ cell proliferation at 1 day; normalized/reduced later; NeuroD^+^ differentiation peaked at 8 days, elevated at 15, baseline by 30 (adult females) * ↑ mBDNF in hippocampus of all groups (peak at 1 day, sustained to 15–30 days, age-/sex-dependent); ECS had no effect on p-ERK1/2 or p-mTOR in adolescents; ↑ p-ERK1/2 at 15 days in adults	[45]
**Neurotransmitter**	Clinical	MDD or patients with depressive episode of bipolar disorder and HC	* 3 times per week * majority recived right unilateral ECT * stimulus intensity was age-based and adjusted per session to achieve 20–25 s of EEG seizure activity	Baseline: * ↔ prefrontal GABA levels, ↔ GABA/NAA ratios and NAA/Cr ratios, ↔ GM content, ↔ Glu/Cr, Gln/Cr, Cho/Cr, GSH/Cr or Glu/GABA ratios,↔ GABA/Cr ratios and NAA/Cr ratios in OCC During ECT: * ↓ NAA/Cr ratios in the PFC Post-ECT: * ↔GABA/Cr levels * ↓ NAA/Cr ratios in the PFC * NAA/Cr ratios decreasing as the number of ECT sessions increased *↔ Glu/Cr, Gln/Cr, Cho/Cr or GSH/Cr levels	[33]
	Clinical	TR-MDD patients	* brief pulse ECT three times per week usually for 12 treatment sessions * right unilateral electrode placement (bitemporal if non-responsive by 6th session or seizures inadequate)	* ↑ basal (T1) epinephrine in responders who later showed ↓ post-first ECT * ↑ epinephrine in all non-responders (ΔT1–T2) * ↓ epinephrine in 7 of 13 responders (ΔT1–T2) * ↓ epinephrine at T3 vs. T1 in responders with initial T1–T2 decrease * ↑ cortisol after first ECT (T1→T2), ↓ at T3 to baseline * ↑ norepinephrine at T2; no further significant changes * ↔ no significant ΔT1–T3 in epinephrine, norepinephrine, or cortisol	[46]
	Clinical	Patients with depression and HC	* right unilateral electrode placement * initial stimulus charge was age-based and increased over sessions to account for rising seizure threshold	Post-ECT: * ↓ tNAA in ACC (~10.6 sessions): 6% (creatine ratio), 3% (water referenced) Follow-up: * tNAA returned to baseline after 6 months * ↑ tNAA at 6-month follow-up: +6% (creatine ratio), +7% (water referenced)	[47]
	Preclinical	Adult female FSL rats and FRL rats with SD rats.	* ECS once daily for 10 consecutive days * earclip electrode placement Parameters: pulse width 0.5 ms, frequency 100 Hz, current 55–70 mA	Post-ECT: * ↓ α2-adrenoceptor binding post-ECS * ↓ α2-adrenoceptor binding by 9% in FC of FSL * ↔ α2-adrenoceptor binding in IN	[48]
	Preclinical	Male SD rats, cLH and SD WT rats	* 6 WT and 6 cLH rats received daily ECS for 5 days * earclip electrode placement Parameters: pulse width 1 ms, frequency 80 Hz, duration 1 ms	Post-ECT: * ↑ glutamate in ECS-treated cLH vs. naive cLH * ↑ glutamate trend in ECS-treated WT vs. naive WT * ↔ glutamine in hippocampus * no baseline differences between untreated cLH and WT in hippocampal glutamate, glutamine, or GABA	[49]
	Preclinical	Male C57BL/6J mice	* ECS was administered 4 times a week for up to 3 weeks * bilateral ECS * earclip electrode placement Parameters: pulse width 0.5 ms, frequency 100 Hz, duration 1 s, current 25 mA	* enhanced synaptic potentiation induced by dopamine, remained during 4 weeks of follow up, but suppressed by D1-like receptor antagonist * graded effect of electrical stimulation	[50]
**Receptor Hypothesis**	Clinical	MDD patients	* ECT sessions 4–13, 3 times per week * right unilateral ECT (bilateral approach used in patients with minimal or no improvement from sixth ECT onward)	Baseline: * ↔ no change in 5-HT1A BPND between PET1 and PET2 in any brain region Post-ECT: * ↓ 5-HT1A BPND (PET2 vs. PET3), with a large cortical cluster (436 cm^3^) showing reductions, peaking in ACC (including sgACC), OFC, IN, hippocampus, and amygdala * no regions showed significant ↑ 5-HT1A BPND post-ECT * no significant effects of sex, ECT laterality, anticonvulsants, treatment outcome, age, or session number on 5-HT1A BPND changes * no hemispheric differences, No correlation between 5-HT1A BPND changes and HAM-D score changes	[51]
	Clinical	TR-MDD patients	* three times a week * right unilateral ECT * seizure threshold was determined during the first session; subsequent treatments used three times this threshold, with 10–20% adjustments to elicit ≥ 20 s seizures measured by single-strip EEG	Post-ECT: * ↓ 5-HT2 receptor binding in bilateral OC, medial PC (peak: LG), limbic cortex (peak: right PHG), bilateral PFC (peak: right inferomedial PFC) * reduction in 5-HT2 binding in right medial PFC, right LG, and right PHG showed trend-level correlation with HRSD improvement	[52]
	Clinical	MDD patients and HC	* 6–7 bilateral ECTs, 2–3 per week * bifrontal electrode placement	* ↓ 5-HT1A BP in midbrain by 32% in MDD patients before ECT vs. controls * ↓ 5-HT1A BP in midbrain raphe by 31% in MDD patients after ECT vs. controls * ↔ no significant change in 5-HT1A BP in MDD patients pre- vs. post-ECT	[53]
	Preclinical	Adult male rhesus monkeys	* ESC administered twice weekly for 3 weeks * bitemporal electrode placement Parameters: pulse width 0.5 ms, frequency 70 Hz, current 0.9 mA	* ↓ [^18^F] setoperone binding potential at 24 h and 1 week post-treatment, ↔ at 4–6 weeks * ↓ 5-HT2 binding in all regions at 24 h and 1 week post-ECS, returning to baseline by 4–6 weeks * ↓ 5-HT2 binding observed after 2–3 ECS treatments in 3 of 5 animals at 24 h post-treatment across most brain regions	[54]
	Preclinical	Male rhesus monkeys	* ECT was administered twice a week over a 3-week period * bilateral temporal electrode placement Parameters: pulse width 0.5 ms, frequency 70 Hz, current 0.9 mA	Post-ECT: * ↑ MP and DTBZ binding in all striatal regions; returned toward baseline by 6–8 weeks * ↑ SCH23390 binding in striatum at 24–48 h post-ECT; ↔ at later time points	[55]
	Preclinical	SD rats	* 10 or five consecutive ECS treatments (one every other day) * earclip electrode placement Parameters: pulse width 500 ms, frequency 50 Hz, duration 1 s, current 100 mA	Post-10 ECT: * ↑ GluR-A (Ser831) by +68% in whole homogenate (+50% in TIF) * ↑ NR2B (Ser1303) by +72% in homogenate (+78% in TIF) * ↔ in phosphorylation of NR1 (Ser896) or total protein levels of any glutamate receptor subunits Post-5 ECS: * ↔ in phosphorylation or protein levels. * Effects in Triton Insoluble Fraction (TIF) confirm synaptic localization of phosphorylation changes	[56]
**Cytokine (Inflammation)**	Clinical	MDD patients and HC	* 1–7 ECT session (3.7 on average) * bilateral electrode placement * initial stimulus was ~5× patient’s age, adjusted for demographics and medications, and modified during ECT based on seizure quality	Post-ECT: * ↑ IL-1A (transient) * ↑ IL-6 (↑ at 3 h & 6 h, ↓ by 24 h) * ↔ IL-1RA	[57]
	Clinical	Patients with severe TR depressive episode	* 2–3 ECT sessions per week * right unilateral brief pulse ECT, bilateral in case of non-responsiveness * seizure threshold was titrated at the first session, with dose increases if seizures were inadequate or no clinical response occurred	Post-ECT (CSF): * ↔ IL-6, neopterin, sCD14, sCD163, MIF, MCP-1 * baseline ↑ sCD14 predicted ↓ HDRS scores * Δ MIF differed between remitters vs. non-remitters	[58]
	Clinical	MDD patients	* total ECT sessions: 10.4 ± 3.6, 3 times per week with a brief pulse Parameters: pulse width 0.5–1 s, frequency 20–50 Hz	Post-ECT: * ↑ IL-6 vs. baseline * ↓ IL-6 (baseline) from first to final ECT in remitters only * ↔ IL-1RA * ↔ IL levels and ECT parameters	[59]
	Preclinical	Biozzi ABH mice (Envigo)	* earclip electrode placement Parameters: pulse width 0.5 ms, frequency 100 Hz, duration 1 s, current charging at 2 mA, starting at 8 Ma	* ↔ spinal microglia count in naïve mice * ↔ microglia count with ECS pre-treatment, but → altered morphology & ↑ RARα pathway activation in response to LPS	[60]
	Preclinical	Male SD rats	* once daily ESC for 10 days * earclip electrode placement Parameters: current 55 mA, duration 0.3 s	* ↑ BrdU+ cells (~2×) in mPFC after 10 daily ECS vs. sham * ↔ BrdU+ cells differentiating into astrocytes in FC * ↑ Rip^+^ (oligodendrocytes) in FC post-ECS * ↑ endothelial cell number after ECS	[61]
	Preclinical	Biozzi mice with first relapse of EAE	* ECS initiation at first day of clinical signs and 4 additional ECS sessions on alternating days * ECS was applied with twice the average threshold	* ↓ T cell infiltration (−59%) and IBA1^+^ microglia/macrophages (−44%) in SC WM after ECS * ↔ oligodendrocyte numbers between groups * ↑ NG2^+^ cells in control EAE (3.8× vs. naive), marginal ↑ in ECS-treated EAE	[62]

Note: Differences in outcome measures across studies (e.g., BDNF source and timing, cytokine type, imaging technique, and ECT protocol) may contribute to observed inconsistencies and should be interpreted in context. Also, findings from preclinical ECS models should be interpreted with caution due to interspecies physiological differences and variability in ECS protocols compared to clinical ECT procedures. Abbreviations: electroconvulsive therapy—ECT; treatment-resistant schizophrenia—TRS; treatment-resistant depression—TRD; healthy controls—HC; Doublecortin—DCX; Cluster of differentiation 81—CD81; neuron-derived extracellular vesicles—NDEVs; Extracellular signal-regulated kinase 1—ERK1; Cyclic AMP response element-binding protein—CREB; v-akt murine thymoma viral oncogene homolog 1—AKT1; Nuclear receptor subfamily 3, group C, member 1—NR3C1; Insulin-like growth factor 1—IGF1; brain-derived neurotrophic factor—BDNF; electroconvulsive shock—ECS; wildtype—WT; Early growth response protein 3—Egr3; dentate gyrus—DG; 5-bromo-2′-deoxyuridine—BrdU; Neurogenic differentiation factor—NeuroD; Mature BDNF—mBDNF; Phosphorylated ERK1/2—p-ERK1/2; Phosphorylated mechanistic target of rapamycin—p-mTOR; major depressive disorder—MDD; gamma-aminobutyric acid—GABA; N-acetylaspartate—NAA; creatine—Cr; gray matter—GM; glutamate—Glu; glutamine—Gln; choline—Cho; glutathione—GSH; orbitofrontal cortex—OFC; insula—IN; prefrontal cortex—PFC; Basal nucleus—Basal; anterior cingulate cortex—ACC; Subgenual anterior cingulate cortex—sgACC; occipital cortex—OC; parietal cortex—PC; Lingual gyrus—LG; Parahippocampal gyrus—PHG; Hamilton Rating Scale for Depression—HAM-D; Hamilton Rating Scale for Depression—HRSD; binding potential—BP; dopamine transporter—DTBZ; Total N-acetylaspartate—tNAA; Fluorine-18—^18^F; Flinders-sensitive line—FSL; Flinders-resistant line—FRL; Sprague–Dawley—SD; Congenital learned helplessness—cLH; Dopamine receptor antagonist—D1-like receptor antagonist; Glutamate receptor A—GluR-A; N-methyl-D-aspartate receptor subunit 2B—NR2B; NMDA receptor subunit 1—NR1; Triton-insoluble fraction—TIF; Serine—Ser; interleukin—IL; Interleukin 1 receptor antagonist—IL-1RA; cerebrospinal fluid—CSF; Soluble CD14—sCD14; Soluble CD163—sCD163; macrophage migration inhibitory factor—MIF; Monocyte chemoattractant protein-1—MCP-1; 5-hydroxytryptamine 1A—5-HT1A; 5-hydroxytryptamine 2—5-HT2; positron emission tomography—PET; Radioligand SCH23390—SCH23390; Methylphenidate—MP; alpha-2 adrenoceptor—α2-adrenoceptor; Receptor alpha—RARα; lipopolysaccharide—LPS; Experimental autoimmune encephalomyelitis—EAE; Spinal cord white matter—SC WM; Ionized calcium-binding adapter molecule 1—IBA1; Nerve/glial antigen 2—NG2; Medial prefrontal cortex—mPFC; frontal cortex—FC.

**Table 2:** Summary of neurotransmitter receptor changes following ECT in clinical and preclinical studies.

Neurotransmitter	Receptor/Target	Study	Brain Region(s)/Blood Components	ECT Effect	Ref.
Serotonin	5HT1A	Clinical	subgenual part of ACC, OFC, AMY, hippocampus, IN midbrain raphe	↓ ↔	[51] [53]
		Preclinical	CA3c DG	↓ ↑	[79]
	5HT2A	Clinical	all cortical areas with changes slightly more prominent in the right hemisphere	↓	[52]
		Preclinical	cingulate and frontoparietal cortex, FC cortical areas	↑ ↓	[79] [52,54]
Dopamine	D1	Preclinical	hippocampal mossy fiber (MF)-CA3 excitatory synapse striatum	↑ ↑	[50] [55]
	D2	Clinical	ACC	↓	[80]
		Preclinical	striatum	↔	[55]
	D3	Preclinical	D3 receptor mRNA and binding shell of nucleus accumbens D3 receptor mRNA islands of Calleja	↑	[81]
Norepinephrine	α2-adrenoceptors	Clinical	platelet α2-adrenoceptor numbers leukocyte α2-adrenoceptor densities	↓ ↑	[82]
		Preclinical	cortical regions and amygdaloid regions	↓	[48]
	NE	Clinical	plasma NE plasma NE plasma level in patients responding to ECT	↑ ↓ ↓	[83] [84] [46]
		Preclinical	presynaptic release of [^3^H] norepinephrine from rat cortical vesicular preparation	↔	[85]
Glutamate	Glu (overall)	Clinical	ACC and connected prefrontal and subcortical centers left hippocampus and right hippocampus the subgenual ACC left ACC	↑ ↓ ↑ ↑	[86] [72] [87]
		Preclinical	hippocampus	↑	[88]

Note: Differences in outcome measures across studies (e.g., BDNF source and timing, cytokine type, imaging technique, and ECT protocol) may contribute to observed inconsistencies and should be interpreted in context. Also, findings from preclinical ECS models should be interpreted with caution due to interspecies physiological differences and variability in ECS protocols compared to clinical ECT procedures. Abbreviations: ACC—anterior cingulate cortex, OFC—orbitofrontal cortex, AMY—amygdala, IN—insula, CA3c—Cornu Ammonis 3 region c, DG—dentate gyrus, FC—frontal cortex, MF-CA3—mossy fiber to CA3 synapse, 5HT1A—5-hydroxytryptamine receptor 1A, 5HT2A—5-hydroxytryptamine receptor 2A, D1—dopamine receptor D1, D2—dopamine receptor D2, D3—dopamine receptor D3, NE—norepinephrine, Glu—glutamate.

**Table 3:** Summary of clinical and preclinical studies investigating structural and functional brain changes following ECT.

Study Type	Subjects	ECT Protocol	Brain Region	Main Findings	Ref.
Clinical	Depressive patients	* twice a week with a constant-current brief-pulse device * mostly right unilateral ECT	Hippocampus	* ↑ bilateral hippocampal volume one week post-ECT; not detectable at 6 months.	[113]
Clinical	Depressive patients	* right unilaterally or bilaterally	Hippocampus and Amygdala	* ↑ whole gray matter (particularly right-sided); not correlated with outcomes.	[114]
Clinical	Patients with unipolar depression vs. HC	* right unilateral electrode placement, later bilateral due to insufficient response	Right hippocampus and Amygdala	* ↑ gray matter volume increased post-ECT; clinical outcomes not assessed.	[115]
Clinical	Depressive patients	* twice a week, bitemporally with a brief pulse	Hippocampus	* ↑ hippocampal volumes; linked to decrease in cognitive functioning.	[116]
Clinical	MDD patients vs. HC	* mostly right unilateral	White matter tracts (DTI)	* ↑ FA, ↓ reduced RD and MD—improved fiber integrity.	[117]
Clinical	Depressive patients vs. HC	* mostly right unilateral	DMN, CEN (functional networks)	* ↓ DMN hyperconnectivity and ↑ CEN connectivity; linked to improvement.	[118]
Preclinical	Rodents and non-human primates	* brief pulse, bilateral frontotemporal electrode placement, 3 times weekly, 4 weeks * earclip electrode placement	DG (hippocampus)	* ↑ progenitor cell proliferation; * ↑ BrdU+ cell increase.	[36,64,119,120]
Preclinical	Male Sprague–Dawley rats	* bilateral ECS via moistened pads on spring-loaded earclip electrodes	Hippocampus and choroid plexus	* ↑ BDNF and VEGF genes expression; associated with synaptic plasticity and recovery.	[121]
Preclinical	Male Wistar rats	* once daily for 10 days * earclip electrodes	Hippocampus (CA1 and CA3 regions)	* ↑ spine density in CA1 neurons in non-stressed animals; * ↔ CA3c spine densities.	[122]

Note: Differences in outcome measures across studies (e.g., BDNF source and timing, cytokine type, imaging technique, and ECT protocol) may contribute to observed inconsistencies and should be interpreted in context. Also, findings from preclinical ECS models should be interpreted with caution due to interspecies physiological differences and variability in ECS protocols compared to clinical ECT procedures. Abbreviations: ECT—electroconvulsive therapy, HC—healthy control, MDD—major depressive disorder, DTI—Diffusion Tensor Imaging, FA—Fractional Anisotropy, RD—Radial Diffusivity, MD—Mean Diffusivity, DMN—default mode network, CEN—central executive network, DG—dentate gyrus, BrdU+—bromodeoxyuridine-positive cells, ECS—electroconvulsive shock, BDNF—brain-derived neurotrophic factor, VEGF—vascular endothelial growth factor, CA1—Cornu Ammonis Region 1, CA3—Cornu Ammonis Region 3.

## References

[1] Marx W., Penninx B.W., Solmi M., Furukawa T.A., Firth J., Carvalho A.F., Berk M. (2023). Major depressive disorder. Nat. Rev. Dis. Primers.

[2] Institute for Health Metrics and Evaluation (2019). Global Burden of Disease Study 2017 (GBD 2017) Data Resources [Internet].

[3] GBD 2019 Mental Disorders Collaborators (2022). Global, regional, and national burden of 12 mental disorders in 204 countries and territories, 1990–2019: A systematic analysis for the global burden of disease study 2019. Lancet Psychiatry.

[4] Mathers C. (2008). The Global Burden of Disease: 2004 Update.

[5] Abe Y., Erchinger V.J., Ousdal O.T., Oltedal L., Tanaka K.F., Takamiya A. (2024). Neurobiological mechanisms of electroconvulsive therapy for depression: Insights into hippocampal volumetric increases from clinical and preclinical studies. J. Neurochem..

[6] Rybak Y.E., Lai K.S., Ramasubbu R., Vila-Rodriguez F., Blumberger D.M., Chan P., Delva N., Giacobbe P., Gosselin C., Kennedy S.H. (2021). Treatment-resistant major depressive disorder: Canadian expert consensus on definition and assessment. Depress. Anxiety.

[7] McIntyre R.S., Alsuwaidan M., Baune B.T., Berk M., Demyttenaere K., Goldberg J.F., Gorwood P., Ho R., Kasper S., Kennedy S.H. (2023). Treatment-resistant depression: Definition, prevalence, detection, management, and investigational interventions. World J. Psychiatry.

[8] McIntyre R.S., Soczynska J.K., Cha D.S., Woldeyohannes H.O., Dale R.S., Alsuwaidan M.T., Gallaugher L.A., Mansur R.B., Muzina D.J., Carvalho A. (2015). The prevalence and illness characteristics of DSM-5-defined “mixed feature specifier” in adults with major depressive disorder and bipolar disorder: Results from the International Mood Disorders Collaborative Project. J. Affect. Disord..

[9] Espinoza R.T., Kellner C.H. (2022). Electroconvulsive therapy. N. Engl. J. Med..

[10] Spaans H.P., Sienaert P., Bouckaert F., van den Berg J.F., Verwijk E., Kho K.H., Stek M.L., Kok R.M. (2015). Speed of remission in elderly patients with depression: Electroconvulsive therapy v. medication. Br. J. Psychiatry.

[11] Kellner C.H., Fink M., Knapp R., Petrides G., Husain M., Rummans T., Mueller M., Bernstein H., Rasmussen K., O’Connor K. (2005). Relief of expressed suicidal intent by ECT: A consortium for research in ECT study. Am. J. Psychiatry.

[12] Trifu S., Sevcenco A., Stănescu M., Drăgoi A.M., Cristea M.B. (2021). Efficacy of electroconvulsive therapy as a potential first-choice treatment in treatment-resistant depression. Exp. Ther. Med..

[13] The UK ECT Review Group (2003). Efficacy and safety of electroconvulsive therapy in depressive disorders: A systematic review and meta-analysis. Lancet.

[14] Kritzer M.D., Peterchev A.V., Camprodon J.A. (2023). Electroconvulsive therapy: Mechanisms of action, clinical considerations, and future directions. Harv. Rev. Psychiatry.

[15] McClintock S.M., Brandon A.R., Husain M.M., Jarrett R.B. (2011). A systematic review of the combined use of electroconvulsive therapy and psychotherapy for depression. J. ECT.

[16] Chen J.J., Zhao L.B., Liu Y.Y., Fan S.H., Xie P. (2017). Comparative efficacy and acceptability of electroconvulsive therapy versus repetitive transcranial magnetic stimulation for major depression: A systematic review and multiple-treatments meta-analysis. Behav. Brain Res..

[17] Kumar S., Mulsant B.H., Liu A.Y., Blumberger D.M., Daskalakis Z.J., Rajji T.K. (2016). Systematic review of cognitive effects of electroconvulsive therapy in late-life depression. Am. J. Geriatr. Psychiatry.

[18] McCall W.V., Lisanby S.H., Rosenquist P.B., Dooley M., Husain M.M., Knapp R.G., Petrides G., Rudorfer M.V., Young R.C., McClintock S.M. (2018). Effects of continuation electroconvulsive therapy on quality of life in elderly depressed patients: A randomized clinical trial. J. Psychiatr. Res..

[19] Liang C.S., Chung C.H., Tsai C.K., Chien W.C. (2017). In-hospital mortality among electroconvulsive therapy recipients: A 17-year nationwide population-based retrospective study. Eur. Psychiatr..

[20] Liang C.S., Chung C.H., Ho P.S., Tsai C.K., Chien W.C. (2018). Superior anti-suicidal effects of electroconvulsive therapy in unipolar disorder and bipolar depression. Bipolar Disord..

[21] Peterchev A.V., Rosa M.A., Deng Z.D., Prudic J., Lisanby S.H. (2010). Electroconvulsive therapy stimulus parameters: Rethinking dosage. J. ECT.

[22] Ryan K.M., McLoughlin D.M. (2019). From Molecules to Mind: Mechanisms of Action of Electroconvulsive Therapy. Focus Am. J. Psychiatry.

[23] Wiedemann L., Trumm S., Bajbouj M., Grimm S., Aust S. (2023). The influence of electroconvulsive therapy on reconsolidation of autobiographical memories: A retrospective quasi-experimental study in patients with depression. Int. J. Clin. Health Psychol..

[24] Miller E. (1967). Psychological theories of ECT: A review. Br. J. Psychiatry.

[25] Cameron D.E. (1960). Production of differential amnesia as a factor in the treatment of schizophrenia. Compr. Psychiatry.

[26] Netto C.A., Izquierdo I. (1984). Amnesia as a major side effect of electroconvulsive shock: The possible involvement of hypothalamic opioid systems. Braz. J. Med. Biol. Res..

[27] Bassa A., Sagués T., Porta-Casteràs D., Serra P., Martínez-Amorós E., Palao D.J., Cano M., Cardoner N. (2021). The neurobiological basis of cognitive side effects of electroconvulsive therapy: A systematic review. Brain Sci..

[28] Neuhaus A.H., Gallinat J., Bajbouj M., Reischies F.M. (2005). Interictal slow-wave focus in left medial temporal lobe during bilateral electroconvulsive therapy. Neuropsychobiology.

[29] Sackeim H.A., Prudic J., Devanand D.P., Kiersky J.E., Fitzsimons L., Moody B.J., McElhiney M.C., Coleman E.A., Settembrino J.M. (1993). Effects of stimulus intensity and electrode placement on the efficacy and cognitive effects of electroconvulsive therapy. N. Engl. J. Med..

[30] Sienaert P., Vansteelandt K., Demyttenaere K., Peuskens J. (2010). Randomized comparison of ultra-brief bifrontal and unilateral electroconvulsive therapy for major depression: Cognitive side-effects. J. Affect. Disord..

[31] Farzan F., Boutros N.N., Blumberger D.M., Daskalakis Z.J. (2014). What does the electroencephalogram tell us about the mechanisms of action of ECT in major depressive disorders?. J. ECT.

[32] Duthie A.C., Perrin J.S., Bennett D.M., Currie J., Reid I.C. (2015). Anticonvulsant mechanisms of electroconvulsive therapy and relation to therapeutic efficacy. J. ECT.

[33] Knudsen M.K., Near J., Blicher A.B., Videbech P., Blicher J.U. (2019). Magnetic resonance (MR) spectroscopic measurement of γ-aminobutyric acid (GABA) in major depression before and after electroconvulsive therapy. Acta Neuropsychiatr..

[34] Deng Z.D., Robins P.L., Regenold W., Rohde P., Dannhauer M., Lisanby S.H. (2024). How electroconvulsive therapy works in the treatment of depression: Is it the seizure, the electricity, or both?. Neuropsychopharmacology.

[35] Altar C.A., Laeng P., Jurata L.W., Brockman J.A., Lemire A., Bullard J., Bukhman Y.V., Young T.A., Charles V., Palfreyman M.G. (2004). Electroconvulsive seizures regulate gene expression of distinct neurotrophic signaling pathways. J. Neurosci..

[36] Perera T.D., Coplan J.D., Lisanby S.H., Lipira C.M., Arif M., Carpio C., Spitzer G., Santarelli L., Scharf B., Hen R. (2007). Antidepressant-induced neurogenesis in the hippocampus of adult nonhuman primates. J. Neurosci..

[37] Haskett R.F. (2014). Electroconvulsive therapy’s mechanism of action: Neuroendocrine hypotheses. J. ECT.

[38] Eşel E., Baştürk M., Kula M., Reyhancan M., Turan M.T., Sofuoğlu S. (2003). Effects of electroconvulsive therapy on pituitary hormones in depressed patients. Klin. Psikofarmakol. Bülteni.

[39] Burgese D.F., Bassitt D.P. (2015). Variation of plasma cortisol levels in patients with depression after treatment with bilateral electroconvulsive therapy. Trends Psychiatry Psychother..

[40] Shahin O., Gohar S.M., Ibrahim W., El-Makawi S.M., Fakher W., Taher D.B., Abdel Samie M., Khalil M.A., Saleh A.A. (2022). Brain-Derived neurotrophic factor (BDNF) plasma level increases in patients with resistant schizophrenia treated with electroconvulsive therapy (ECT). Int. J. Psychiatry Clin..

[41] Xie X.H., Xu S.X., Yao L., Chen M.M., Zhang H., Wang C., Nagy C., Liu Z. (2024). Altered in vivo early neurogenesis traits in patients with depression: Evidence from neuron-derived extracellular vesicles and electroconvulsive therapy. Brain Stimul..

[42] Schurgers G., Walter S., Pishva E., Guloksuz S., Peerbooms O., Incio L.R., Arts B.M., Kenis G., Rutten B.P. (2022). Longitudinal alterations in mRNA expression of the BDNF neurotrophin signaling cascade in blood correlate with changes in depression scores in patients undergoing electroconvulsive therapy. Eur. Neuropsychopharmacol..

[43] Ramnauth A.D., Maynard K.R., Kardian A.S., Phan B.N., Tippani M., Rajpurohit S., Hobbs J.W., Page S.C., Jaffe A.E., Martinowich K. (2022). Induction of Bdnf from promoter I following electroconvulsive seizures contributes to structural plasticity in neurons of the piriform cortex. Brain Stimul..

[44] Meyers K.T., Damphousse C.C., Ozols A.B., Campbell J.M., Newbern J.M., Hu C., Marrone D.F., Gallitano A.L. (2023). Serial electroconvulsive Seizure alters dendritic complexity and promotes cellular proliferation in the mouse dentate gyrus; a role for Egr3. Brain Stimul..

[45] Ledesma-Corvi S., García-Fuster M.J. (2023). Electroconvulsive seizures regulate various stages of hippocampal cell genesis and mBDNF at different times after treatment in adolescent and adult rats of both sexes. Front. Mol. Neurosci..

[46] Pollak C., Maier H.B., Moschny N., Jahn K., Bleich S., Frieling H., Neyazi A. (2021). Epinephrine levels decrease in responders after electroconvulsive therapy. J. Neural Transm..

[47] Erchinger V.J., Craven A.R., Ersland L., Oedegaard K.J., Bartz-Johannessen C.A., Hammar Å., Haavik J., Riemer F., Kessler U., Oltedal L. (2023). Electroconvulsive therapy triggers a reversible decrease in brain N-acetylaspartate. Front. Psychiatry.

[48] Lillethorup T.P., Iversen P., Fontain J., Wegener G., Doudet D.J., Landau A.M. (2015). Electroconvulsive shocks decrease α2-adrenoceptor binding in the Flinders rat model of depression. Eur. Neuropsychopharmacol..

[49] Biedermann S., Weber-Fahr W., Zheng L., Hoyer C., Vollmayr B., Gass P., Ende G., Sartorius A. (2012). Increase of hippocampal glutamate after electroconvulsive treatment: A quantitative proton MR spectroscopy study at 9.4 T in an animal model of depression. World J. Biol. Psychiatry.

[50] Kobayashi K., Imoto Y., Yamamoto F., Kawasaki M., Ueno M., Segi-Nishida E., Suzuki H. (2017). Rapid and lasting enhancement of dopaminergic modulation at the hippocampal mossy fiber synapse by electroconvulsive treatment. J. Neurophysiol..

[51] Lanzenberger R., Baldinger P., Hahn A., Ungersboeck J., Mitterhauser M., Winkler D., Micskei Z., Stein P., Karanikas G., Wadsak W. (2013). Global decrease of serotonin-1A receptor binding after electroconvulsive therapy in major depression measured by PET. Mol. Psychiatry.

[52] Yatham L.N., Liddle P.F., Lam R.W., Zis A.P., Stoessl A.J., Sossi V., Adam M.J., Ruth T.J. (2010). Effect of electroconvulsive therapy on brain 5-HT2 receptors in major depression. Br. J. Psychiatry.

[53] Saijo T., Takano A., Suhara T., Arakawa R., Okumura M., Ichimiya T., Ito H., Okubo Y. (2010). Effect of electroconvulsive therapy on 5-HT1A receptor binding in patients with depression: A PET study with [11C] WAY 100635. Int. J. Neuropsychopharmacol..

[54] Strome E.M., Clark C.M., Zis A.P., Doudet D.J. (2005). Electroconvulsive shock decreases binding to 5-HT2 receptors in nonhuman primates: An in vivo positron emission tomography study with [18F] setoperone. Biol. Psychiatry.

[55] Landau A.M., Chakravarty M.M., Clark C.M., Zis A.P., Doudet D.J. (2011). Electroconvulsive therapy alters dopamine signaling in the striatum of non-human primates. Neuropsychopharmacology.

[56] Fumagalli F., Pasini M., Sartorius A., Scherer R., Racagni G., Riva M.A., Gass P. (2010). Repeated electroconvulsive shock (ECS) alters the phosphorylation of glutamate receptor subunits in the rat hippocampus. Int. J. Neuropsychopharmacol..

[57] Lehtimäki K., Keränen T., Huuhka M., Palmio J., Hurme M., Leinonen E., Peltola J. (2008). Increase in plasma proinflammatory cytokines after electroconvulsive therapy in patients with depressive disorder. J. ECT.

[58] Yoshimura R., Mitoma M., Sugita A., Hori H., Okamoto T., Umene W., Ueda N., Nakamura J. (2007). Effects of paroxetine or milnacipran on serum brain-derived neurotrophic factor in depressed patients. Prog. Neuro-Psychopharmacol. Biol. Psychiatry.

[59] Järventausta K., Sorri A., Kampman O., Björkqvist M., Tuohimaa K., Hämäläinen M., Moilanen E., Leinonen E., Peltola J., Lehtimäki K. (2017). Changes in interleukin-6 levels during electroconvulsive therapy may reflect the therapeutic response in major depression. Acta Psychiatr. Scand..

[60] Goldfarb S., Fainstein N., Ganz T., Vershkov D., Lachish M., Ben-Hur T. (2021). Electric neurostimulation regulates microglial activation via retinoic acid receptor α signaling. Brain Behav. Immun..

[61] Madsen T.M., Yeh D.D., Valentine G.W., Duman R.S. (2005). Electroconvulsive seizure treatment increases cell proliferation in rat frontal cortex. Neuropsychopharmacology.

[62] Goldfarb S., Fainstein N., Ben-Hur T. (2020). Electroconvulsive stimulation attenuates chronic neuroinflammation. JCI Insight.

[63] Bouckaert F., Sienaert P., Obbels J., Dols A., Vandenbulcke M., Stek M., Bolwig T. (2014). ECT: Its brain enabling effects: A review of electroconvulsive therapy–induced structural brain plasticity. J. ECT.

[64] Madsen T.M., Treschow A., Bengzon J., Bolwig T.G., Lindvall O., Tingström A. (2000). Increased neurogenesis in a model of electroconvulsive therapy. Biol. Psychiatry.

[65] Kato N. (2009). Neurophysiological mechanisms of electroconvulsive therapy for depression. Neurosci. Res..

[66] Willard S.S., Koochekpour S. (2013). Glutamate, glutamate receptors, and downstream signaling pathways. Int. J. Biol. Sci..

[67] Gilbert J.R., Yarrington J.S., Wills K.E., Nugent A.C., Zarate C.A. (2018). Glutamatergic signaling drives ketamine-mediated response in depression: Evidence from dynamic causal modeling. Int. J. Neuropsychopharmacol..

[68] Zhou W., Wang N., Yang C., Li X.M., Zhou Z.Q., Yang J.J. (2014). Ketamine-induced antidepressant effects are associated with AMPA receptors-mediated upregulation of mTOR and BDNF in rat hippocampus and prefrontal cortex. Eur. Psychiatry.

[69] Li N., Lee B., Liu R.J., Banasr M., Dwyer J.M., Iwata M., Li X.Y., Aghajanian G., Duman R.S. (2010). mTOR-dependent synapse formation underlies the rapid antidepressant effects of NMDA antagonists. Science.

[70] Sanacora G., Zarate C.A., Krystal J.H., Manji H.K. (2008). Targeting the glutamatergic system to develop novel, improved therapeutics for mood disorders. Nat. Rev. Drug Discov..

[71] De Jager J.E., Boesjes R., Roelandt G.H., Koliaki I., Sommer I.E., Schoevers R.A., Nuninga J.O. (2024). Shared effects of electroconvulsive shocks and ketamine on neuroplasticity: A systematic review of animal models of depression. Neurosci. Biobehav. Rev..

[72] Njau S., Joshi S.H., Espinoza R., Leaver A.M., Vasavada M., Marquina A., Woods R.P., Narr K.L. (2017). Neurochemical correlates of rapid treatment response to electroconvulsive therapy in patients with major depression. J. Psychiatry Neurosci..

[73] Baldinger P., Lotan A., Frey R., Kasper S., Lerer B., Lanzenberger R. (2014). Neurotransmitters and electroconvulsive therapy. J. ECT.

[74] Cojocaru A.M., Vasile A.I., Trifu S.C. (2024). Neurobiological mechanisms and therapeutic impact of electroconvulsive therapy (ECT). Rom. J. Morphol. Embryol..

[75] Landau A.M., Phan J.A., Iversen P., Lillethorup T.P., Simonsen M., Wegener G., Jakobsen S., Doudet D.J. (2015). Decreased in vivo α2 adrenoceptor binding in the Flinders Sensitive Line rat model of depression. Neuropharmacology.

[76] Lillethorup T.P., Iversen P., Wegener G., Doudet D.J.M., Landau A.M. (2015). α2-adrenoceptor binding in Flinders-sensitive line compared with Flinders-resistant line and Sprague-Dawley rats. Acta Neuropsyciatr..

[77] Dai X., Zhang R., Deng N., Tang L., Zhao B. (2024). Anesthetic Influence on Electroconvulsive Therapy: A Comprehensive Review. Neuropsychiatr. Dis. Treat..

[78] Markianos M., Hatzimanolis J., Lykouras L. (2002). Relationship between prolactin responses to ECT and dopaminergic and serotonergic responsivity in depressed patients. Eur. Arch. Psychiatry Clin. Neurosci..

[79] Burnet P.W.J., Mead A., Eastwood S.L., Lacey K., Harrison P.J., Sharp T. (1995). Repeated ECS differentially affects rat brain 5-HT1A and 5-HT2A receptor expression. Neuroreport.

[80] Saijo T., Takano A., Suhara T., Arakawa R., Okumura M., Ichimiya T., Ito H., Okubo Y. (2010). Electroconvulsive therapy decreases dopamine D2 receptor binding in the anterior cingulate in patients with depression: A controlled study using positron emission tomography with radioligand [11C] FLB 457. J. Clin. Psychiatry.

[81] Lammers C.H., Diaz J., Schwartz J.C., Sokoloff P. (2000). Selective increase of dopamine D3 receptor gene expression as a common effect of chronic antidepressant treatments. Mol. Psychiatry.

[82] Werstiuk E.S., Coote M., Griffith L., Shannon H., Steiner M. (1996). Effects of electroconvulsive therapy on peripheral adrenoceptors, plasma, noradrenaline, MHPG and cortisol in depressed patients. Br. J. Psychiatry.

[83] Mann J.J., Manevitz A.Z., Chen J.S., Johnson K.S., Adelsheimer E.F., Azima-Heller R., Massina A., Wilner P.J. (1990). Acute effects of single and repeated electroconvulsive therapy on plasma catecholamines and blood pressure in major depressive disorder. Psychiatry Res..

[84] Kelly C.B., Cooper S.J. (1997). Plasma noradrenaline response to electroconvulsive therapy in depressive illness. Br. J. Psychiatry.

[85] Ebstein R.P., Lerer B., Shlaufman M., Belmaker R.H. (1983). The effect of repeated electroconvulsive shock treatment and chronic lithium feeding on the release of norepinephrine from rat cortical vesicular preparations. Cell. Mol. Neurobiol..

[86] Zhang J., Narr K.L., Woods R.P., Phillips O.R., Alger J.R., Espinoza R.T. (2013). Glutamate normalization with ECT treatment response in major depression. Mol. Psychiatry.

[87] Pfleiderer B., Michael N., Erfurth A., Ohrmann P., Hohmann U., Wolgast M., Fiebich M., Arolt V., Heindel W. (2003). Effective electroconvulsive therapy reverses glutamate/glutamine deficit in the left anterior cingulum of unipolar depressed patients. Psychiatry Res. Neuroimaging.

[88] Dong J., Min S., Wei K., Li P., Cao J., Li Y. (2010). Effects of electroconvulsive therapy and propofol on spatial memory and glutamatergic system in hippocampus of depressed rats. J. ECT.

[89] Burnet P.W., Sharp T., LeCorre S.M., Harrison P.J. (1999). Expression of 5-HT receptors and the 5-HT transporter in rat brain after electroconvulsive shock. Neurosci. Lett..

[90] Newman M.E., Gur E., Shapira B., Lerer B. (1998). Neurochemical mechanisms of action of ECS: Evidence from in vivo studies. J. ECT.

[91] Chaput Y., de Montigny C., Blier P. (1991). Presynaptic and postsynaptic modifications of the serotonin system by long-term administration of antidepressant treatments. An in vivo electrophysiologic study in the rat. Neuropsychopharmacology.

[92] Gray J.A., Roth B.L. (2001). Paradoxical trafficking and regulation of 5-HT2A receptors by agonists and antagonists. Brain Res. Bull..

[93] Meyer J.H., Kapur S., Eisfeld B., Brown G.M., Houle S., DaSilva J., Wilson A.A., Rafi-Tari S., Mayberg H.S., Kennedy S.H. (2001). The effect of paroxetine on 5-HT2A receptors in depression: An [18F] setoperone PET imaging study. Am. J. Psychiatry.

[94] Nikisch G., Mathé A.A. (2008). CSF monoamine metabolites and neuropeptides in depressed patients before and after electroconvulsive therapy. Eur. Psychiatr..

[95] Okamoto T., Yoshimura R., Ikenouchi-Sugita A., Hori H., Umene-Nakano W., Inoue Y., Ueda N., Nakamura J. (2008). Efficacy of electroconvulsive therapy is associated with changing blood levels of homovanillic acid and brain-derived neurotrophic factor (BDNF) in refractory depressed patients: A pilot study. Prog. Neuro-Psychopharmacol. Biol. Psychiatry.

[96] Huuhka K., Anttila S., Huuhka M., Hietala J., Huhtala H., Mononen N., Lehtimäki T., Leinonen E. (2008). Dopamine 2 receptor C957T and catechol-o-methyltransferase Val158Met polymorphisms are associated with treatment response in electroconvulsive therapy. Neurosci. Lett..

[97] Ambade V., Arora M.M., Singh P., Somani B.L., Basannar D. (2009). Adrenaline, noradrenaline and dopamine level estimation in depression: Does it help?. Med. J. Armed Forces India.

[98] El Mansari M., Guiard B.P., Chernoloz O., Ghanbari R., Katz N., Blier P. (2010). Relevance of norepinephrine–dopamine interactions in the treatment of major depressive disorder. CNS Neurosci. Ther..

[99] Sartorius A., Mahlstedt M.M., Vollmayr B., Henn F.A., Ende G. (2007). Elevated spectroscopic glutamate/γ-amino butyric acid in rats bred for learned helplessness. Neuroreport.

[100] Hashimoto K., Sawa A., Iyo M. (2007). Increased levels of glutamate in brains from patients with mood disorders. Biol. Psychiatry.

[101] Hasler G., van der Veen J.W., Tumonis T., Meyers N., Shen J., Drevets W.C. (2007). Reduced prefrontal glutamate/glutamine and γ-aminobutyric acid levels in major depression determined using proton magnetic resonance spectroscopy. Arch. Gen. Psychiatry.

[102] Lau A., Tymianski M. (2010). Glutamate receptors, neurotoxicity and neurodegeneration. Pflug. Arch. Eur. J. Physiol..

[103] Kupcova I., Danisovic L., Grgac I., Harsanyi S. (2022). Anxiety and depression: What do we know of neuropeptides?. Behav. Sci..

[104] Kask A., Harro J., von Hörsten S., Redrobe J.P., Dumont Y., Quirion R. (2002). The neurocircuitry and receptor subtypes mediating anxiolytic-like effects of neuropeptide Y. Neurosci. Biobehav. Rev..

[105] Ozsoy S., Eker O.O., Abdulrezzak U. (2016). The effects of antidepressants on neuropeptide Y in patients with depression and anxiety. Pharmacopsychiatry.

[106] Stenfors C., Mathé A.A., Theodorsson E. (1994). Repeated electroconvulsive stimuli: Changes in neuropeptide Y, neurotensin and tachykinin concentrations in time. Prog. Neuropsychopharmacol. Biol. Psychiatry.

[107] Mikkelsen J.D., Woldbye D.P. (2006). Accumulated increase in neuropeptide Y and somatostatin gene expression of the rat in response to repeated electroconvulsive stimulation. J. Psychiatr. Res..

[108] Stengaard-Pedersen K., Schou M. (1986). Opioid receptors in the brain of the rat following chronic treatment with desipramine and electroconvulsive shock. Neuropharmacology.

[109] Weizman A., Gil-Ad I., Grupper D., Tyano S., Laron Z. (1987). The effect of acute and repeated electroconvulsive treatment on plasma β-endorphin, growth hormone, prolactin and cortisol secretion in depressed patients. Psychopharmacology.

[110] Kolb B., Whishaw I.Q. (1998). Brain plasticity and behavior. Annu. Rev. Psychol..

[111] Zatorre R.J., Fields R.D., Johansen-Berg H. (2012). Plasticity in gray and white: Neuroimaging changes in brain structure during learning. Nat. Neurosci..

[112] Puderbaugh M., Emmady P.D. (2023). Neuroplasticity.

[113] Bouckaert F., Dols A., Emsell L., De Winter F.L., Vansteelandt K., Claes L., Sunaert S., Stek M., Sienaert P., Vandenbulcke M. (2016). Relationship between hippocampal volume, serum BDNF, and depression severity following electroconvulsive therapy in late-life depression. Neuropsychopharmacology.

[114] Sartorius A., Demirakca T., Böhringer A., von Hohenberg C.C., Aksay S.S., Bumb J.M., Kranaster L., Nickl-Jockschat T., Grözinger M., Thomann P.A. (2019). Electroconvulsive therapy induced gray matter increase is not necessarily correlated with clinical data in depressed patients. Brain Stimul..

[115] Camilleri J.A., Hoffstaedter F., Zavorotny M., Zöllner R., Wolf R.C., Thomann P., Redlich R., Opel N., Dannlowski U., Groezinger M. (2020). Electroconvulsive therapy modulates grey matter increase in a hub of an affect processing network. Neuroimage Clin..

[116] van Oostrom I., van Eijndhoven P., Butterbrod E., van Beek M.H., Janzing J., Donders R., Schene A., Tendolkar I. (2018). Decreased cognitive functioning after electroconvulsive therapy is related to increased hippocampal volume: Exploring the role of brain plasticity. J. ECT.

[117] Lyden H., Espinoza R.T., Pirnia T., Clark K., Joshi S.H., Leaver A.M., Woods R.P., Narr K.L. (2014). Electroconvulsive therapy mediates neuroplasticity of white matter microstructure in major depression. Transl. Psychiatry.

[118] Abbott C.C., Lemke N.T., Gopal S., Thoma R.J., Bustillo J., Calhoun V.D., Turner J.A. (2013). Electroconvulsive therapy response in major depressive disorder: A pilot functional network connectivity resting state FMRI investigation. Front. Psychiatry.

[119] Malberg J.E., Eisch A.J., Nestler E.J., Duman R.S. (2000). Chronic antidepressant treatment increases neurogenesis in adult rat hippocampus. J. Neurosci..

[120] Scott B.W., Wojtowicz J.M., Burnham W.M. (2000). Neurogenesis in the dentate gyrus of the rat following electroconvulsive shock seizures. Exp. Neurol..

[121] Newton S.S., Collier E.F., Hunsberger J., Adams D., Terwilliger R., Selvanayagam E., Duman R.S. (2003). Gene profile of electroconvulsive seizures: Induction of neurotrophic and angiogenic factors. J. Neurosci..

[122] Müller H.K., Orlowski D., Bjarkam C.R., Wegener G., Elfving B. (2015). Potential roles for Homer1 and Spinophilin in the preventive effect of electroconvulsive seizures on stress-induced CA3c dendritic retraction in the hippocampus. Eur. Neuropsychopharmacol..

[123] Joshi S.H., Espinoza R.T., Pirnia T., Shi J., Wang Y., Ayers B., Leaver A., Woods R.P., Narr K.L. (2016). Structural plasticity of the hippocampus and amygdala induced by electroconvulsive therapy in major depression. Biol. Psychiatry.

[124] Nordanskog P., Dahlstrand U., Larsson M.R., Larsson E.M., Knutsson L., Johanson A. (2010). Increase in hippocampal volume after electroconvulsive therapy in patients with depression: A volumetric magnetic resonance imaging study. J. ECT.

[125] Jorgensen A., Magnusson P., Hanson L.G., Kirkegaard T., Benveniste H., Lee H., Svarer C., Mikkelsen J.D., Fink-Jensen A., Knudsen G.M. (2016). Regional brain volumes, diffusivity, and metabolite changes after electroconvulsive therapy for severe depression. Acta Psychiatr. Scand..

[126] Argyelan M., Lencz T., Kang S., Ali S., Masi P.J., Moyett E., Joanlanne A., Watson P., Sanghani S., Petrides G. (2021). ECT-induced cognitive side effects are associated with hippocampal enlargement. Transl. Psychiatry.

[127] O’Donovan S., Kennedy M., Guinan B., O’Mara S., McLoughlin D.M. (2012). A comparison of brief pulse and ultrabrief pulse electroconvulsive stimulation on rodent brain and behaviour. Prog. Neuropsychopharmacol. Biol. Psychiatry.

[128] Ousdal O.T., Brancati G.E., Kessler U., Erchinger V., Dale A.M., Abbott C., Oltedal L. (2022). The neurobiological effects of electroconvulsive therapy studied through magnetic resonance: What have we learned, and where do we go?. Biol. Psychiatry.

[129] Pirnia T., Joshi S.H., Leaver A.M., Vasavada M., Njau S., Woods R.P., Espinoza R., Narr K.L. (2016). Electroconvulsive therapy and structural neuroplasticity in neocortical, limbic and paralimbic cortex. Transl. Psychiatry.

[130] Sorri A., Järventausta K., Kampman O., Lehtimäki K., Björkqvist M., Tuohimaa K., Hämäläinen M., Moilanen E., Leinonen E. (2018). Effect of electroconvulsive therapy on brain-derived neurotrophic factor levels in patients with major depressive disorder. Brain Behav..

[131] Kyeremanteng C., MacKay J.C., James J.S., Kent P., Cayer C., Anisman H., Merali Z. (2014). Effects of electroconvulsive seizures on depression-related behavior, memory and neurochemical changes in Wistar and Wistar–Kyoto rats. Prog. Neuropsychopharmacol. Biol. Psychiatry.

[132] Luo J., Min S., Wei K., Cao J., Wang B., Li P., Dong J., Liu Y. (2015). Behavioral and molecular responses to electroconvulsive shock differ between genetic and environmental rat models of depression. Psychiatry Res..

[133] Shiraishi-Yamaguchi Y., Furuichi T. (2007). The Homer family proteins. Genome Biol..

[134] Hu J.H., Park J.M., Park S., Xiao B., Dehoff M.H., Kim S., Hayashi T., Schwarz M.K., Huganir R.L., Seeburg P.H. (2010). Homeostatic scaling requires group I mGluR activation mediated by Homer1a. Neuron.

[135] Serchov T., Clement H.W., Schwarz M.K., Iasevoli F., Tosh D.K., Idzko M., Jacobson K.A., de Bartolomeis A., Normann C., Biber K. (2015). Increased signaling via adenosine A1 receptors, sleep deprivation, imipramine, and ketamine inhibit depressive-like behavior via induction of Homer1a. Neuron.

[136] Wagner K.V., Hartmann J., Labermaier C., Häusl A.S., Zhao G., Harbich D., Schmid B., Wang X.D., Santarelli S., Kohl C. (2015). Homer1/mGluR5 activity moderates vulnerability to chronic social stress. Neuropsychopharmacology.

[137] Li M., Yao X., Sun L., Zhao L., Xu W., Zhao H., Zhao F., Zou X., Cheng Z., Li B. (2020). Effects of electroconvulsive therapy on depression and its potential mechanism. Front. Psychol..

[138] Pang Y., Wei Q., Zhao S., Li N., Li Z., Lu F., Pang J., Zhang R., Wang K., Chu C. (2022). Enhanced default mode network functional connectivity links with electroconvulsive therapy response in major depressive disorder. J. Affect. Disord..

[139] Jelovac A., Kolshus E., McLoughlin D.M. (2013). Relapse following successful electroconvulsive therapy for major depression: A meta-analysis. Neuropsychopharmacology.

[140] Miller A.H., Raison C.L. (2016). The role of inflammation in depression: From evolutionary imperative to modern treatment target. Nat. Rev. Immunol..

[141] Nordenskjöld A., von Knorring L., Ljung T., Carlborg A., Brus O., Engström I. (2013). Continuation electroconvulsive therapy with pharmacotherapy versus pharmacotherapy alone for prevention of relapse of depression: A randomized controlled trial. J. ECT.

[142] Sackeim H.A., Haskett R.F., Mulsant B.H., Thase M.E., Mann J.J., Pettinati H.M., Greenberg R.M., Crowe R.R., Cooper T.B., Prudic J. (2001). Continuation pharmacotherapy in the prevention of relapse following electroconvulsive therapy: A randomized controlled trial. JAMA.

[143] Freire T.F.V., da Rocha N.S., de Almeida Fleck M.P. (2017). The association of electroconvulsive therapy to pharmacological treatment and its influence on cytokines. J. Psychiatr. Res..

[144] Van Den Bossche M.J., Emsell L., Dols A., Vansteelandt K., De Winter F.L., Van den Stock J., Sienaert P., Stek M.L., Bouckaert F., Vandenbulcke M. (2019). Hippocampal volume change following ECT is mediated by rs699947 in the promotor region of VEGF. Transl. Psychiatry.

[145] Levy M.J., Boulle F., Steinbusch H.W., van den Hove D.L., Kenis G., Lanfumey L. (2018). Neurotrophic factors and neuroplasticity pathways in the pathophysiology and treatment of depression. Psychopharmacology.

[146] Pardossi S., Fagiolini A., Cuomo A. (2024). Variations in BDNF and Their Role in the Neurotrophic Antidepressant Mechanisms of Ketamine and Esketamine: A Review. Int. J. Mol. Sci..

[147] Ito M., Seki T., Liu J., Nakamura K., Namba T., Matsubara Y., Suzuki T., Arai H. (2010). Effects of repeated electroconvulsive seizure on cell proliferation in the rat hippocampus. Synapse.

[148] Ricken R., Adli M., Lange C., Krusche E., Stamm T.J., Gaus S., Koehler S., Nase S., Bschor T., Richter C. (2013). Brain-derived neurotrophic factor serum concentrations in acute depressive patients increase during lithium augmentation of antidepressants. J. Clin. Psychopharmacol..

[149] Mikoteit T., Beck J., Eckert A., Hemmeter U., Brand S., Bischof R., Holsboer-Trachsler E., Delini-Stula A. (2014). High baseline BDNF serum levels and early psychopathological improvement are predictive of treatment outcome in major depression. Psychopharmacology.

[150] Nase S., Köhler S., Jennebach J., Eckert A., Schweinfurth N., Gallinat J., Lang U.E., Kühn S. (2018). Role of serum brain derived neurotrophic factor and central n-acetylaspartate for clinical response under antidepressive pharmacotherapy. Neurosignals.

[151] Yoshimura R., Kishi T., Hori H., Katsuki A., Sugita-Ikenouchi A., Umene-Nakano W., Atake K., Iwata N., Nakamura J. (2014). Serum levels of brain-derived neurotrophic factor at 4 weeks and response to treatment with SSRIs. Psychiatry Investig..

[152] Tadić A., Wagner S., Schlicht K.F., Peetz D., Borysenko L., Dreimüller N., Hiemke C., Lieb K. (2011). The early non-increase of serum BDNF predicts failure of antidepressant treatment in patients with major depression: A pilot study. Prog. Neuro-Psychopharmacol. Biol. Psychiatry.

[153] Dreimüller N., Schlicht K.F., Wagner S., Peetz D., Borysenko L., Hiemke C., Lieb K., Tadić A. (2012). Early reactions of brain-derived neurotrophic factor in plasma (pBDNF) and outcome to acute antidepressant treatment in patients with Major Depression. Neuropharmacology.

[154] Karege F., Perret G., Bondolfi G., Schwald M., Bertschy G., Aubry J.M. (2002). Decreased serum brain-derived neurotrophic factor levels in major depressed patients. Psychiatry Res..

[155] Haile C.N., Murrough J.W., Iosifescu D.V., Chang L.C., Al Jurdi R.K., Foulkes A., Iqbal S., Mahoney J.J., De La Garza R., Charney D.S. (2014). Plasma brain derived neurotrophic factor (BDNF) and response to ketamine in treatment-resistant depression. Int. J. Neuropsychopharmacol..

[156] Piccinni A., Del Debbio A., Medda P., Bianchi C., Roncaglia I., Veltri A., Zanello S., Massimetti E., Origlia N., Domenici L. (2009). Plasma Brain-Derived Neurotrophic Factor in treatment-resistant depressed patients receiving electroconvulsive therapy. Eur. Neuropsychopharmacol..

[157] Maffioletti E., Gennarelli M., Gainelli G., Bocchio-Chiavetto L., Bortolomasi M., Minelli A. (2019). BDNF genotype and baseline serum levels in relation to electroconvulsive therapy effectiveness in treatment-resistant depressed patients. J. ECT.

[158] Ryan K.M., Dunne R., McLoughlin D.M. (2018). BDNF plasma levels and genotype in depression and the response to electroconvulsive therapy. Brain Stimul..

[159] Fernandes B., Gama C.S., Massuda R., Torres M., Camargo D., Kunz M., Belmonte-de-Abreu P.S., Kapczinski F., de Almeida Fleck M.P., Lobato M.I. (2009). Serum brain-derived neurotrophic factor (BDNF) is not associated with response to electroconvulsive therapy (ECT): A pilot study in drug resistant depressed patients. Neurosci. Lett..

[160] van Zutphen E.M., Rhebergen D., van Exel E., Oudega M.L., Bouckaert F., Sienaert P., Vandenbulcke M., Stek M., Dols A. (2019). Brain-derived neurotrophic factor as a possible predictor of electroconvulsive therapy outcome. Transl. Psychiatry.

[161] Vanicek T., Kranz G.S., Vyssoki B., Fugger G., Komorowski A., Höflich A., Saumer G., Milovic S., Lanzenberger R., Eckert A. (2019). Acute and subsequent continuation electroconvulsive therapy elevates serum BDNF levels in patients with major depression. Brain Stimul..

[162] Zelada M.I., Garrido V., Liberona A., Jones N., Zúñiga K., Silva H., Nieto R.R. (2023). Brain-derived neurotrophic factor (BDNF) as a predictor of treatment response in major depressive disorder (MDD): A systematic review. Int. J. Mol. Sci..

[163] Maynard K.R., Hobbs J.W., Rajpurohit S.K., Martinowich K. (2018). Electroconvulsive seizures influence dendritic spine morphology and BDNF expression in a neuroendocrine model of depression. Brain Stimul..

[164] Ryan K.M., O’Donovan S.M., McLoughlin D.M. (2013). Electroconvulsive stimulation alters levels of BDNF-associated microRNAs. Neurosci. Lett..

[165] Pu J., Liu Y., Gui S., Tian L., Xu S., Song X., Zhong X., Chen Y., Chen X., Yu Y. (2020). Vascular endothelial growth factor in major depressive disorder, schizophrenia, and bipolar disorder: A network meta-analysis. Psychiatry Res..

[166] Sharma A.N., Soares J.C., Carvalho A.F., Quevedo J. (2016). Role of trophic factors GDNF, IGF-1 and VEGF in major depressive disorder: A comprehensive review of human studies. J. Affect. Disord..

[167] Minelli A., Zanardini R., Abate M., Bortolomasi M., Gennarelli M., Bocchio-Chiavetto L. (2011). Vascular Endothelial Growth Factor (VEGF) serum concentration during electroconvulsive therapy (ECT) in treatment resistant depressed patients. Prog. Neuro-Psychopharmacol. Biol. Psychiatry.

[168] Minelli A., Maffioletti E., Bortolomasi M., Conca A., Zanardini R., Rillosi L., Abate M., Giacopuzzi M., Maina G., Gennarelli M. (2014). Association between baseline serum vascular endothelial growth factor levels and response to electroconvulsive therapy. Acta Psychiatr. Scand..

[169] Grønli O., Stensland G.Ø., Wynn R., Olstad R. (2009). Neurotrophic factors in serum following ECT: A pilot study. World J. Biol. Psychiatry.

[170] Zhang X., Zhang Z., Sha W., Xie C., Xi G., Zhou H., Zhang Y. (2009). Electroconvulsive therapy increases glial cell-line derived neurotrophic factor (GDNF) serum levels in patients with drug-resistant depression. Psychiatry Res..

[171] Angelucci F., Aloe L., Jiménez-Vasquez P., Mathé A.A. (2002). Electroconvulsive stimuli alter the regional concentrations of nerve growth factor, brain-derived neurotrophic factor, and glial cell line-derived neurotrophic factor in adult rat brain. J. ECT.

[172] Enomoto S., Shimizu K., Nibuya M., Suzuki E., Nagata K., Kondo T. (2017). Activated brain-derived neurotrophic factor/TrkB signaling in rat dorsal and ventral hippocampi following 10-day electroconvulsive seizure treatment. Neurosci. Lett..

[173] Miller A.H., Maletic V., Raison C.L. (2009). Inflammation and its discontents: The role of cytokines in the pathophysiology of major depression. Biol. Psychiatry.

[174] Mössner R., Mikova O., Koutsilieri E., Saoud M., Ehlis A.C., Müller N., Fallgatter A.J., Riederer P. (2007). Consensus paper of the WFSBP Task Force on Biological Markers: Biological markers in depression. World J. Biol. Psychiatry.

[175] Silverman M.N., Macdougall M.G., Hu F., Pace T.W.W., Raison C.L., Miller A.H. (2007). Endogenous glucocorticoids protect against TNF-alpha-induced increases in anxiety-like behavior in virally infected mice. Mol. Psychiatry.

[176] Simen B.B., Duman C.H., Simen A.A., Duman R.S. (2006). TNFα signaling in depression and anxiety: Behavioral consequences of individual receptor targeting. Biol. Psychiatry.

[177] Tyring S., Gottlieb A., Papp K., Gordon K., Leonardi C., Wang A., Lalla D., Woolley M., Jahreis A., Zitnik R. (2006). Etanercept and clinical outcomes, fatigue, and depression in psoriasis: Double-blind placebo-controlled randomised phase III trial. Lancet.

[178] Wang C., Zong S., Cui X., Wang X., Wu S., Wang L., Liu Y., Lu Z. (2023). The effects of microglia-associated neuroinflammation on Alzheimer’s disease. Front. Immunol..

[179] Yirmiya R., Rimmerman N., Reshef R. (2015). Depression as a microglial disease. Trends Neurosci..

[180] Kronfol Z.A., Lemay L., Nair M.P., Kluger M.J. (1990). Electroconvulsive Therapy Increases Plasma Levels of Interleukin-6a. Neuropeptides and Immunopeptides: Messengers in a Neuroinunune Axis.

[181] Hestad K.A., Tønseth S., Støen C.D., Ueland T., Aukrust P. (2003). Raised plasma levels of tumor necrosis factor α in patients with depression: Normalization during electroconvulsive therapy. J. ECT.

[182] Kranaster L., Hoyer C., Aksay S.S., Bumb J.M., Müller N., Zill P., Schwarz M.J., Sartorius A. (2018). Antidepressant efficacy of electroconvulsive therapy is associated with a reduction of the innate cellular immune activity in the cerebrospinal fluid in patients with depression. World J. Biol. Psychiatry.

[183] Fluitman S.B., Heijnen C.J., Denys D.A., Nolen W.A., Balk F.J., Westenberg H.G. (2011). Electroconvulsive therapy has acute immunological and neuroendocrine effects in patients with major depressive disorder. J. Affect. Disord..

[184] Kronfol Z., Nair M.P., Weinberg V., Young E.A., Aziz M. (2002). Acute effects of electroconvulsive therapy on lymphocyte natural killer cell activity in patients with major depression. J. Affect. Disord..

[185] Moschny N., Jahn K., Maier H.B., Khan A.Q., Ballmaier M., Liepach K., Sack M., Skripuletz T., Bleich S., Frieling H. (2020). Electroconvulsive therapy, changes in immune cell ratios, and their association with seizure quality and clinical outcome in depressed patients. Eur. Neuropsychopharmacol..

[186] Donato R. (2001). S100: A multigenic family of calcium-modulated proteins of the EF-hand type with intracellular and extracellular functional roles. Int. J. Biochem. Cell Biol..

[187] Marenholz I., Heizmann C.W., Fritz G. (2004). S100 proteins in mouse and man: From evolution to function and pathology (including an update of the nomenclature). Biochem. Biophys. Res. Commun..

[188] Winningham-Major F., Staecker J.L., Barger S.W., Coats S., Van Eldik L.J. (1989). Neurite extension and neuronal survival activities of recombinant S100 beta proteins that differ in the content and position of cysteine residues. J. Cell Biol..

[189] Arts B., Peters M., Ponds R., Honig A., Menheere P., van Os J. (2006). S100 and impact of ECT on depression and cognition. J. ECT.

[190] Aziz N., Nishanian P., Mitsuyasu R., Detels R., Fahey J.L. (1999). Variables that affect assays for plasma cytokines and soluble activation markers. Clin. Diagn. Lab. Immunol..

[191] Khan M., Baussan Y., Hebert-Chatelain E. (2023). Connecting Dots between Mitochondrial Dysfunction and Depression. Biomolecules.

[192] Mailloux R.J. (2020). An update on mitochondrial reactive oxygen species production. Antioxidants.

[193] Andrés Juan C., Pérez de Lastra J.M., Plou Gasca F.J., Pérez-Lebeña E. (2021). The chemistry of reactive oxygen species (ROS) revisited: Outlining their role in biological macromolecules (DNA, lipids and proteins) and induced pathologies. Int. J. Mol. Sci..

[194] Mattson M.P., Gleichmann M., Cheng A. (2008). Mitochondria in neuroplasticity and neurological disorders. Neuron.

[195] Petschner P., Gonda X., Baksa D., Eszlari N., Trivaks M., Juhasz G., Bagdy G. (2018). Genes linking mitochondrial function, cognitive impairment and depression are associated with endophenotypes serving precision medicine. Neuroscience.

[196] Jiang M., Wang L., Sheng H. (2024). Mitochondria in depression: The dysfunction of mitochondrial energy metabolism and quality control systems. CNS Neurosci. Ther..

[197] Gebara E., Zanoletti O., Ghosal S., Grosse J., Schneider B.L., Knott G., Astori S., Sandi C. (2021). Mitofusin-2 in the nucleus accumbens regulates anxiety and depression-like behaviors through mitochondrial and neuronal actions. Biol. Psychiatry.

[198] Wu T., Huang Y., Gong Y., Xu Y., Lu J., Sheng H., Ni X. (2019). Treadmill exercise ameliorates depression-like behavior in the rats with prenatal dexamethasone exposure: The role of hippocampal mitochondria. Front. Neurosci..

[199] Gong Y., Chai Y., Ding J.H., Sun X.L., Hu G. (2011). Chronic mild stress damages mitochondrial ultrastructure and function in mouse brain. Neurosci. Lett..

[200] Caruso G., Benatti C., Blom J.M., Caraci F., Tascedda F. (2019). The many faces of mitochondrial dysfunction in depression: From pathology to treatment. Front. Pharmacol..

[201] Li W., Zhu L., Chen Y., Zhuo Y., Wan S., Guo R. (2023). Association between mitochondrial DNA levels and depression: A systematic review and meta-analysis. BMC Psychiatry.

[202] Fattal O., Link J., Quinn K., Cohen B.H., Franco K. (2007). Psychiatric comorbidity in 36 adults with mitochondrial cytopathies. CNS Spectr..

[203] Gardner A., Johansson A., Wibom R., Nennesmo I., von Döbeln U., Hagenfeldt L., Hällström T. (2003). Alterations of mitochondrial function and correlations with personality traits in selected major depressive disorder patients. J. Affect. Disord..

[204] Brymer K.J., Fenton E.Y., Kalynchuk L.E., Caruncho H.J. (2018). Peripheral etanercept administration normalizes behavior, hippocampal neurogenesis, and hippocampal reelin and GABAA receptor expression in a preclinical model of depression. Front. Pharmacol..

[205] Wang J., Hodes G.E., Zhang H., Zhang S., Zhao W., Golden S.A., Bi W., Menard C., Kana V., Leboeuf M. (2018). Epigenetic modulation of inflammation and synaptic plasticity promotes resilience against stress in mice. Nat. Commun..

[206] Sequeira A., Rollins B., Magnan C., van Oven M., Baldi P., Myers R.M., Barchas J.D., Schatzberg A.F., Watson S.J., Akil H. (2015). Mitochondrial mutations in subjects with psychiatric disorders. PLoS ONE.

[207] Kasahara T., Kubota M., Miyauchi T., Ishiwata M., Kato T. (2008). A marked effect of electroconvulsive stimulation on behavioral aberration of mice with neuron-specific mitochondrial DNA defects. PLoS ONE.

[208] Búrigo M., Roza C.A., Bassani C., Fagundes D.A., Rezin G.T., Feier G., Dal-Pizzol F., Quevedo J., Streck E.L. (2006). Effect of electroconvulsive shock on mitochondrial respiratory chain in rat brain. Neurochem. Res..

[209] Gandhi S., Abramov A.Y. (2012). Mechanism of oxidative stress in neurodegeneration. Oxid. Med. Cell. Longev..

[210] Yoshikawa T., You F. (2024). Oxidative stress and bio-regulation. Int. J. Mol. Sci..

[211] Jazvinšćak Jembrek M., Oršolić N., Karlović D., Peitl V. (2023). Flavonols in action: Targeting oxidative stress and neuroinflammation in major depressive disorder. Int. J. Mol. Sci..

[212] Gadoth N., Göbel H.H. (2011). Oxidative Stress in Applied Basic Research and Clinical Practice.

[213] Bakunina N., Pariante C.M., Zunszain P.A. (2015). Immune mechanisms linked to depression via oxidative stress and neuroprogression. Immunology.

[214] Maes M., Galecki P., Chang Y.S., Berk M. (2011). A review on the oxidative and nitrosative stress (O&NS) pathways in major depression and their possible contribution to the (neuro) degenerative processes in that illness. Prog. Neuro-Psychopharmacol. Biol. Psychiatry.

[215] Bhatt S., Nagappa A.N., Patil C.R. (2020). Role of oxidative stress in depression. Drug Discov. Today.

[216] Ait Tayeb A.E.K., Poinsignon V., Chappell K., Bouligand J., Becquemont L., Verstuyft C. (2023). Major depressive disorder and oxidative stress: A review of peripheral and genetic biomarkers according to clinical characteristics and disease stages. Antioxidants.

[217] Jiménez-Fernández S., Gurpegui M., Garrote-Rojas D., Gutiérrez-Rojas L., Carretero M.D., Correll C.U. (2022). Oxidative stress parameters and antioxidants in adults with unipolar or bipolar depression versus healthy controls: Systematic review and meta-analysis. J. Affect. Disord..

[218] Atagün M.İ., Canbek Ö.A. (2022). A systematic review of the literature regarding the relationship between oxidative stress and electroconvulsive therapy. Alpha Psychiatry.

[219] Bader M., Abdelwanis M., Maalouf M., Jelinek H.F. (2024). Detecting depression severity using weighted random forest and oxidative stress biomarkers. Sci. Rep..

[220] Barichello T., Bonatto F., Feier G., Martins M.R., Moreira J.C.F., Dal-Pizzol F., Izquierdo I., Quevedo J. (2004). No evidence for oxidative damage in the hippocampus after acute and chronic electroshock in rats. Brain Res..

[221] Şahin Ş., Aybastı Ö., Elboğa G., Altındağ A., Tamam L. (2017). Major depresyonda elektrokonvulsif terapinin oksidatif metabolizma üzerine etkisi. Çukurova Med. J..

[222] Lv Q., Hu Q., Zhang W., Huang X., Zhu M., Geng R., Cheng X., Bao C., Wang Y., Zhang C. (2020). Disturbance of oxidative stress parameters in treatment-resistant bipolar disorder and their association with electroconvulsive therapy response. Int. J. Neuropsychopharmacol..

[223] Karayağmurlu E., Elboğa G., Şahin Ş.K., Karayağmurlu A., Taysı S., Ulusal H., Altındağ A. (2022). Effects of electroconvulsive therapy on nitrosative stress and oxidative DNA damage parameters in patients with a depressive episode. Int. J. Psychiatry.

[224] Barichello T., Bonatto F., Agostinho F.R., Reinke A., Moreira J.C.F., Dal-Pizzol F., Izquierdo I., Quevedo J. (2004). Structure-related oxidative damage in rat brain after acute and chronic electroshock. Neurochem. Res..

[225] Župan G., Pilipović K., Hrelja A., Peternel S. (2008). Oxidative stress parameters in different rat brain structures after electroconvulsive shock-induced seizures. Prog. Neuro-Psychopharmacol. Biol. Psychiatry.

[226] Eraković V., Župan G., Varljen J., Radošević S., Simonić A. (2000). Electroconvulsive shock in rats: Changes in superoxide dismutase and glutathione peroxidase activity. Mol. Brain Res..

[227] Nielsen B., Cejvanovic V., Wörtwein G., Hansen A.R., Marstal K.K., Weimann A., Bjerring P.N., Dela F., Poulsen H.E., Jørgensen M.B. (2019). Increased oxidation of RNA despite reduced mitochondrial respiration after chronic electroconvulsive stimulation of rat brain tissue. Neurosci. Lett..

[228] Hollville E., Romero S.E., Deshmukh M. (2019). Apoptotic cell death regulation in neurons. FEBS Lett..

[229] Erekat N.S. (2022). Apoptosis and its therapeutic implications in neurodegenerative diseases. Clin. Anat..

[230] Lucassen P.J., Heine V.M., Muller M.B., van der Beek E.M., Wiegant V.M., De Kloet E.R., Joels M., Fuchs E., Swaab D.F., Czeh B. (2006). Stress, depression and hippocampal apoptosis. CNS Neurol. Disord. Drug Targets.

[231] Kondratyev A., Sahibzada N., Gale K. (2001). Electroconvulsive shock exposure prevents neuronal apoptosis after kainic acid-evoked status epilepticus. Mol. Brain Res..

[232] Zarubenko I.I., Yakovlev A.A., Stepanichev M.Y., Gulyaeva N.V. (2005). Electroconvulsive shock induces neuron death in the mouse hippocampus: Correlation of neurodegeneration with convulsive activity. Neurosci. Behav. Physiol..

[233] Sigström R., Göteson A., Joas E., Pålsson E., Liberg B., Nordenskjöld A., Blennow K., Zetterberg H., Landén M. (2024). Blood biomarkers of neuronal injury and astrocytic reactivity in electroconvulsive therapy. Mol. Psychiatry.

[234] McGrory C.L., Ryan K.M., Kolshus E., McLoughlin D.M. (2019). Peripheral blood E2F1 mRNA in depression and following electroconvulsive therapy. Prog. Neuro-Psychopharmacol. Biol. Psychiatry.

[235] Jeon W.J., Kim S.H., Seo M.S., Kim Y., Kang U.G., Juhnn Y.S., Kim Y.S. (2008). Repeated electroconvulsive seizure induces c-Myc down-regulation and Bad inactivation in the rat frontal cortex. Exp. Mol. Med..

